# Discovery of Novel Inhibitors against ALS-Related SOD1(A4V) Aggregation through the Screening of a Chemical Library Using Differential Scanning Fluorimetry (DSF)

**DOI:** 10.3390/ph17101286

**Published:** 2024-09-27

**Authors:** Maria Giannakou, Ifigeneia Akrani, Angeliki Tsoka, Vassilios Myrianthopoulos, Emmanuel Mikros, Constantinos Vorgias, Dimitris G. Hatzinikolaou

**Affiliations:** 1 Biochemistry and Molecular Biology Unit, Department of Biology, National and Kapodistrian University of Athens, 15784 Zografou, Greece; 2 Enzyme and Microbial Biotechnology Unit, Department of Biology, National and Kapodistrian University of Athens, 15784 Zografou, Greece; 3 Laboratory of Pharmaceutical Chemistry, Department of Pharmacy, National and Kapodistrian University of Athens, 15784 Zografou, Greece; iakrani@pharm.uoa.gr (I.A.);

**Keywords:** ALS, SOD1, protein-misfolding diseases, drug discovery, drug repurposing, chemical library scanning, DSF technique

## Abstract

Background: Cu/Zn Superoxide Dismutase 1 (SOD1) is a 32 kDa cytosolic dimeric metalloenzyme that neutralizes superoxide anions into oxygen and hydrogen peroxide. Mutations in SOD1 are associated with ALS, a disease causing motor neuron atrophy and subsequent mortality. These mutations exert their harmful effects through a gain of function mechanism, rather than a loss of function. Despite extensive research, the mechanism causing selective motor neuron death still remains unclear. A defining feature of ALS pathogenesis is protein misfolding and aggregation, evidenced by ubiquitinated protein inclusions containing SOD1 in affected motor neurons. This work aims to identify compounds countering SOD1(A4V) misfolding and aggregation, which could potentially aid in ALS treatment. Methods: The approach employed was in vitro screening of a library comprising 1280 pharmacologically active compounds (LOPAC^®^) in the context of drug repurposing. Using differential scanning fluorimetry (DSF), these compounds were tested for their impact on SOD1(A4V) thermal stability. Results and Conclusions: Dimer stability was the parameter chosen as the criterion for screening, since the dissociation of the native SOD1 dimer is the step prior to its in vitro aggregation. The screening revealed one compound raising protein-ligand T_m_ by 6 °C, eleven inducing a higher second T_m_, suggesting a stabilization effect, and fourteen reducing T_m_ from 10 up to 26 °C, suggesting possible interactions or non-specific binding.

## 1. Introduction

Amyotrophic lateral sclerosis (ALS) is an incurable motor neuron (MN) degenerative disorder [1,2,3,4]. It is the most common form of MN disease with an adult onset and the third most common neurodegenerative disease [5,6]. Half of the patients survive for 3 or more years post-diagnosis, 20% for 5 years or more, and only up to 10% survive for more than 10 years [7]. Its incidence (ca. 1.9 per 100,000/year) and prevalence (ca. 5.2 per 100,000) are relatively uniform in Western countries [8], whereas its incidence is lower in Asia (ca. 0.8 cases 100,000) [2].

Based on whether a patient has a family history of the disease or not, ALS is classified as familial (fALS) or sporadic (sALS), respectively [6,9]. The reported rate for fALS ranges between 5% and 20%, with the variance being attributed to the uncertainty about what constitutes family history, and the fact that gene variants may occasionally result in sALS [3]. Cu/Zn Superoxide Dismutase 1 (SOD1) mutations were identified as causing fALS in 1993 [10], and SOD1 remains the most extensively investigated ALS-related protein, with more than 30 different genes linked to fALS [9,11]. fALS and sALS are clinically indistinguishable [12], conferring one common characteristic; the presence of ubiquitinated skein-like or round cytoplasmic inclusions (inclusion bodies, IBs) [13]. These IBs are SOD1 immunoreactive [6,14,15,16], but this immunoreactivity is controversial in sALS [17,18].

SOD1 is a homodimeric copper (Cu)- and zinc (Zn)-containing cytosolic enzyme with one intramolecular disulfide bridge per subunit, and its main action is to convert toxic superoxide radicals (O_2_^−^) into oxygen (O_2_) and hydrogen peroxide (H_2_O_2_) [6,19,20,21]. SOD1’s enzyme activity depends on the presence of Cu at the active site, while Zn has a structural role [22,23]. SOD1 is able to keep the intra-molecular disulfide bond intact, despite the reducing environment of the cytosol. Two Cu/Zn-loaded and oxidized subunits are held together with hydrophobic contacts [24] that further exhibit a very high dimerization constant (K_d_ ca. 10^−10^ M^−1^). These post-translational modifications (PTMs) endow the protein with high stability [25,26], shifting its unfolding transition for some ALS mutants from body temperature [27] up to 75 °C for the fully mature WT (wild type) enzyme [27,28].

Mutations distort the native conformation of SOD1 and render the protein prone to losing its PTMs and, consequently, its characteristic stability, exerting a large effect on the monomer–dimer equilibrium [6,24]. Mutated SOD1, particularly with mutations like A4V, has a compromised dimer interface that promotes monomerization. These monomers are structurally disturbed, destabilized, and highly prone to aggregation, contributing to ALS pathology by forming toxic intracellular inclusions that damage MNs [6,25,29,30,31,32,33,34,35,36,37]. Thus far, more than 190 ALS-related mutations have been identified in SOD1 (https://alsod.ac.uk/, accessed on 14 June 2024 [38]) (Figure S1). Most of these mutations result in the aberrant folding of SOD1, thereby leading to the formation and accumulation of oligomeric and aggregated species of the protein [22] and/or alterations in enzyme activity [39,40]. ALS-related mutations of SOD1 are scattered through the entire sequence of the SOD1 protein, suggesting an impact on the whole protein [41,42].

One particular mutation, A4V (Alanine at codon 4 changes to Valine), accounts for almost 50% of ALS-related SOD1 mutations in the USA, but is rarer in Europe [43]. SOD1(A4V) provokes a rapid and aggressive form of fALS, reducing the estimated survival time to less than 2 years [44]. The A4V mutation destabilizes SOD1 in both the apo and metalated states [45,46], involving the distortion of the structure of the protein and the exposure of the hydrophobic core, the destruction of the β-barrel structure and the copper-binding site, along with severe structural changes in the dimer interface [47,48]. The exact details of how SOD1 aggregates cause toxicity are still being investigated. However, several consequences of the aggregates include the following: the sequestration of other proteins essential for cellular components [49,50], reductions in the availability of chaperones needed by other proteins [24,49], and the disruption of the ubiquitin–proteasome pathway by impairing its ability to degrade not only the mutant protein itself but also other proteins [24,49].

Despite its severity, ALS still remains incurable [3,6]. The current drugs available for ALS patients, approved by the US Food and Drug Administration, are as follows: (i) riluzole, whose mechanism of action is related to the inhibition of pre-synaptic glutamate release [8]; (ii) edaravone, an antioxidative compound reducing oxidative stress [9,51]; (iii) tofersen, an antisense oligonucleotide that decreases SOD1 protein synthesis [52]; (iv) sodium phenylbutyrate and taurursodiol, which relate to the amelioration of the production of mitochondrial energy [53,54]; and (v) dextromethorphan HBr and quinidine sulfate, which are used as symptomatic treatments for the pseudobulbar affect [55].

ALS is characterized as a rare disease (<50 cases per 100,000 [56]); however, it still constitutes a significant socio-economic burden in terms of healthcare costs [57]. An estimation of ca. 223,000 patients were affected globally in 2015 [58], and projections state that, in 2040, the number of individuals with the disorder will reach ca. 376,000 [57]. Consequently, there is an urgent need to discover effective therapies against ALS. Since protein misfolding and aggregation are considered to be focal events in ALS pathogenesis, therapeutic strategies targeting these processes hold promise [59]. The understanding of SOD1 aggregation kinetics has introduced new possibilities for ALS therapy through the discovery of small molecules that bind and stabilize either (i) the native state of SOD1 or (ii) the intermediate aggregate species (misfolded monomers, low molecular SOD1 aggregates) [6,24,41,60]. Among them, pharmacological chaperones are small-molecular-weight molecules that can bind onto specific proteins and promote proper folding and stability, thereby inhibiting aggregation and/or degradation [61,62]. Since their first description in the early 2000s, [63] pharmacological chaperones have entered the clinical research stage and subsequent practice for rare conformational diseases [64,65], e.g., tafamidis against transthyretin amyloidosis (ATTR), stabilizing the transthyretin (TTR) tetramer [66,67], migalastat, which assists the proper folding of Fabry disease (FD)-related α-galactosidase (GLA) variants [68], etc. Although pharmacological chaperones show promise for ALS treatment, a phase III trial with arimoclomol did not improve outcomes significantly compared to a placebo [69,70]. This underscores the challenges in drug discovery for ALS, given the inherent difficulties in translating preclinical successes into effective clinical treatments due to the complex and heterogeneous nature of the disease.

Drug discovery practice demonstrates that usually only one out of a thousand compounds may be a viable drug option. ALS drug discovery is even more challenging due to the inherent difficulties in identifying compounds that bind to a mixture of transient aggregation species during aggregation, the fact that there are no natural ligands of SOD1 to serve as a molecular scaffold [71], and the heterogenous effects of mutations. In this study, we identify rescuers of SOD1 misfolding by screening a commercially available library, LOPAC^®^ (Library of Pharmacologically Active Compounds), using the calculated melting temperature of the protein, the T_m_ value, as a proxy for protein stability. LOPAC^®^ contains 1280 marketed drugs and well-characterized small molecules displaying lead-like or drug-like properties. To our knowledge, this is the first time that the LOPAC^®^ library has been used in the context of a random in vitro screening against ALS-related SOD1 variants for the discovery of inhibitors of its aggregation (protein–drug interaction). A thermal shift assay (TSA), more commonly described as differential scanning fluorometry (DSF) [72] was chosen for the screening due to its advantages, which include a high sensitivity, low cost, low required protein and ligand concentrations (of a few μM and mM, respectively), and the simple requirement of a real time-polymerase chain reaction (RT-qPCR) machine, which is commonly available in every laboratory [73,74,75]. For these reasons, DSF can be used for the following: buffer optimization for protein purification, stability and crystallization [73,76], stability in the context of mutations or PTMs [60], analyzing the thermodynamic properties of a protein [77], and in the field of PPI [78] for drug discovery [79,80,81].

## 2. Results

### 2.1. Purification and Characterization of Human-SOD1

For the production of both SOD1 forms (WT and A4V), we chose to employ mild overexpression conditions (low T and the use of a medium copy-number vector such as pASK75). Overexpressed proteins reached a consistent yield of around 8–12 mg/mL eluent or −80–100 mg protein per L culture, which is considered satisfactory given the high propensity of SOD1 to aggregate and its associated difficulty to be retained on the Ni beads (Figures S2 and S3, for WT and A4V, respectively).

It is important to evaluate the ratio among the different conformational species of SOD1 and separate the dimeric form of the protein, since it corresponds to the physiological state of the protein, and we hypothesized this conformational status to be the target of the compounds tested in this study. Inevitably, the mutSOD1 dimer is less stable and it has a heightened tendency to monomerize and exist in dynamic mixtures with intermediate misfolded and/or oligomeric species compared to the WT dimer, both in vitro and in vivo [27,48,82]. Thus, following elution from the nickel column, both SOD1s were subjected to gel filtration chromatography on a custom-made calibrated column.

**Figure 1:**
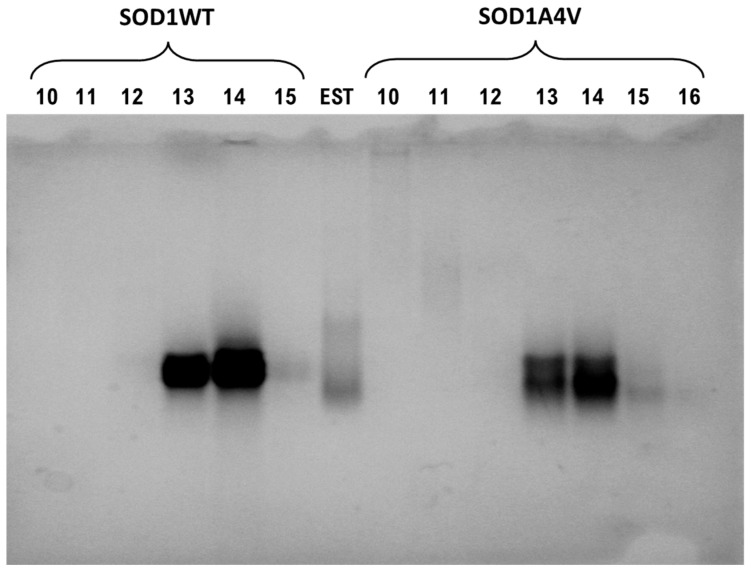
Native-PAGE results for the selected fractions of both SOD1WT (lanes 10–15) and SOD1(A4V) (10–16), aiming to provide an overview of the size of the structures that correspond to the native conformational status of the protein, the dimer (lanes 13 and 14 for both variants). EST corresponds to ESTDZ2, and was chosen to be close in size to the proteins of interest, in order to be used as a standard compound for the identification of the sizes of the proteins analyzed. ESDDZ2 is a globular protein with a molecular weight close to 28 kDa [83], while that of Flag-SOD1-6His is 35.4 kDa.

**Figure 2:**
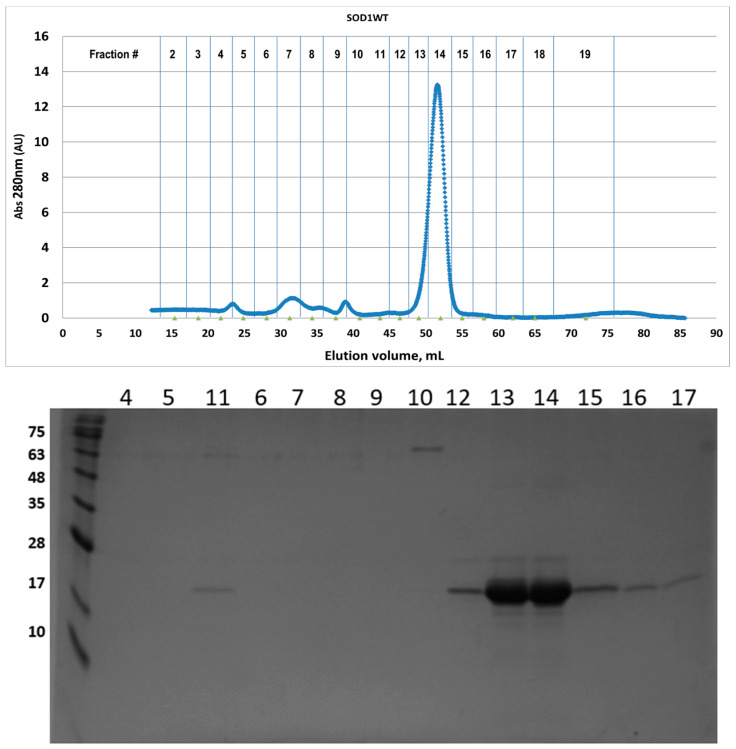
(**Top**) Gel filtration chromatogram of SOD1WT eluted after the first purification step in the NiNTA. Protein was monitored via UV detection (A_280_). Column used: Sephacryl™ S-200 (GE Healthcare, Chicago, IL, USA) with mobile phase TBS buffer pH7.4. (**Bottom**) SDS-PAGE of the selected fractions of purified SOD1WT after gel filtration.

**Figure 3:**
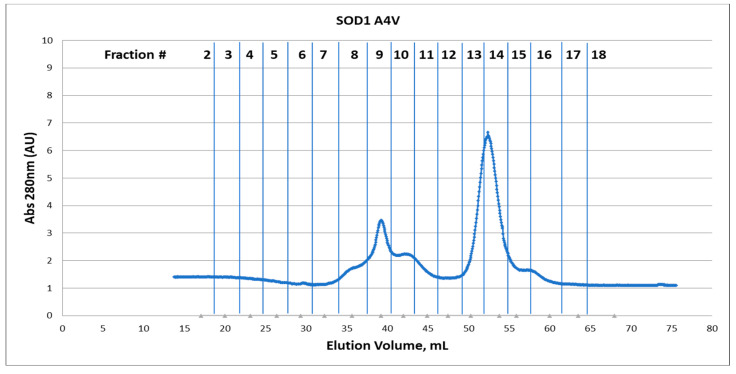

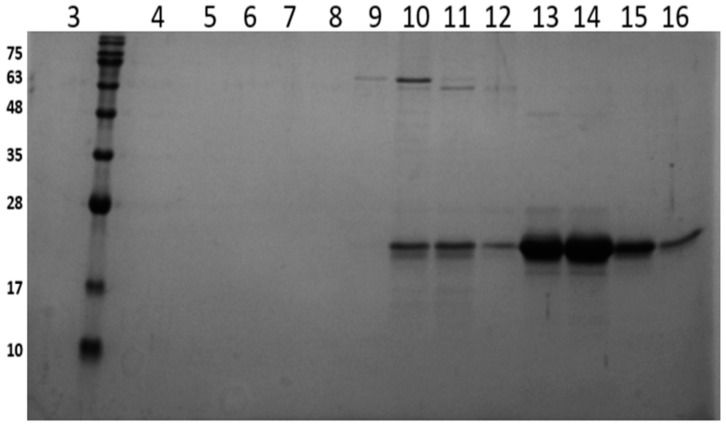
(**Top**) Gel filtration chromatogram of SOD1(A4V) eluted after the first purification step in the NiNTA. Protein was monitored via UV detection (A_280_). Column used: Sephacryl™ S-200 with mobile phase TBS buffer pH7.4. (**Bottom**) SDS-PAGE of the selected fractions of purified SOD1WT after gel filtration.

As seen in Figure 1 and Figure 2, for the WT, the dominating species is the dimeric form, while, for A4V, despite the mild overexpression conditions, this form is significantly decreased (Figure 1 and Figure 3). For the A4V mutant form, it is obvious from the area ratio between the dimeric form and the proceeding peaks corresponding to aggregation species that the dimer state of the protein is noticeably smaller (Figure 1 and Figure 3). A considerable portion of the protein is eluted earlier in the context of a complex and non-separated wide peak (elution fractions 7 to 11; Figure 3), which corresponds to higher molecular weights, as seen from Native-PAGE (Figure 1). These aggregate species also contain an insoluble component, as seen from SDS-PAGE (Figure 3), possibly corresponding to a trimeric species.

**Figure 4:**
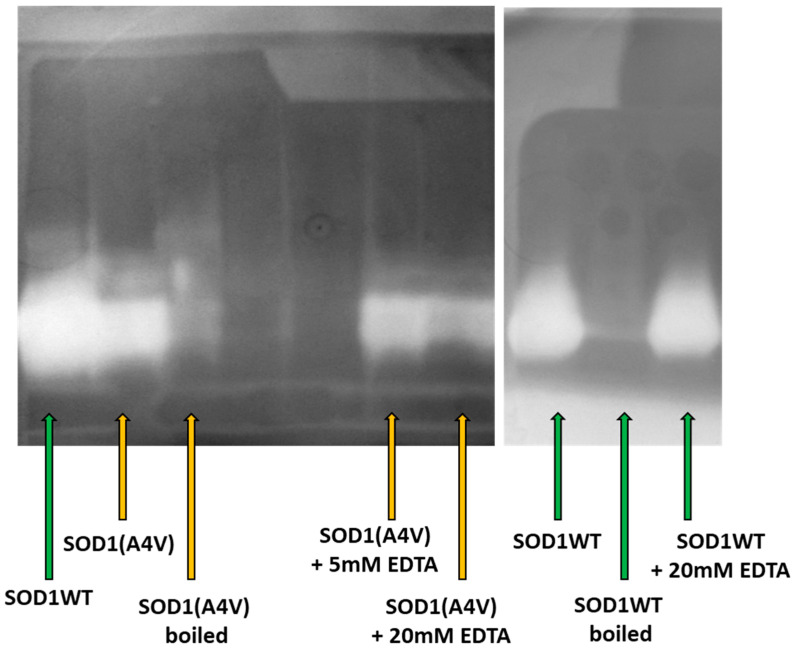
Zymogram performed using the method of riboflavin and B2/NBT. Equimolar quantities of the WT and A4V SOD1 variants were used, in order to estimate the effect of the mutation A4V on the protein. Boiling and the addition of the chelator EDTA were tested in order to estimate the tolerance/stability of each variant. The boiling treatment corresponded to 5 min at 95 °C, while the EDTA treatment corresponded to 15 min incubation at the indicated EDTA concentrations before loading onto the gel occurred.

The activity of the purified SOD1 forms was finally determined via zymogram, using the riboflavin and B2/NBT assay, as described in the Material and Methods section. Based on the zymogram assay, both SOD1 WT and A4V appeared qualitatively active. The analysis of the band intensity after equimolar loading, using the ImageJ software (Version 1.54j), suggested that SOD1(A4V) activity reached almost 87% of the SOD1 WT activity (Figure 4). Furthermore, denaturation at 95 °C led to an almost 90% loss of activity for both variants. Lastly, the presence of an equimolar amount of EDTA chelator reduces the scavenging capacity of SOD1(A4V) by 20%, in contrast to the almost unaffected SOD1 WT activity (Figure 4), suggesting the high stability of the WT enzyme.

#### SOD1 Thermal Stability Characterization Using DSF

During DSF, the protein of interest was heated in the presence/absence of candidate compounds, and the only requirement was the addition of a fluorophore, hydrophobic dye (SYPRO Orange), which bound specifically to the hydrophobic regions of the protein during unfolding and increased its fluorescence. The fluorescence signal was monitored and a sigmoidal curve was extracted, from which the melting temperature (T_m_) of a protein could be extrapolated. T_m_ corresponds to the state wherein the protein was 50% folded and 50% unfolded [73,75].

We employed this technique as a tool for SOD1’s PTM assessment. It is well established that SOD1’s PTMs, including metal incorporation and the formation of the intramolecular disulfide bridge, increase protein stability [27,28]. Adding EDTA, a metal chelator, or DTT, a reducing agent, to break the disulfide linkages was intended to reveal useful information about the quality of the protein overexpressed by our system.

**Figure 5:**
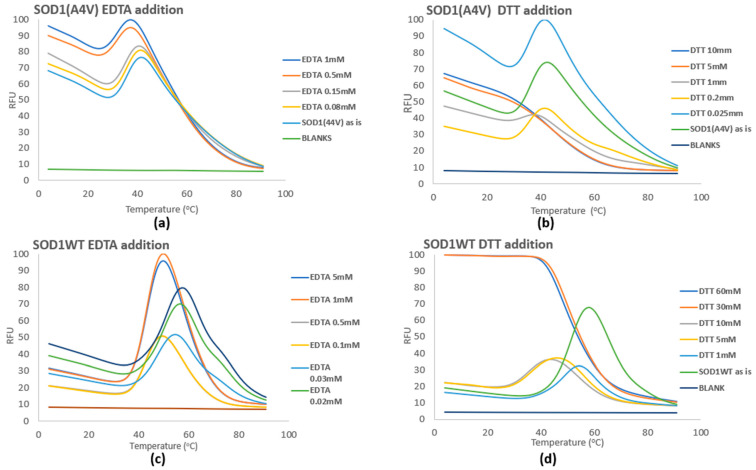
Addition of EDTA and DTT to SOD1(A4V) (**a**,**b**) and SOD1WT (**c**,**d**). SOD1 variants were diluted in the following buffer (elution buffer): 50 mM NaH_2_PO_4_, 300 mM NaCl, and 250 mM imidazole; pH 8.0. RFU corresponds to the normalized fluorescence from triplicate samples.

Both EDTA and DTT, when added to the SOD1 protein variants, exerted a dose-dependent effect, causing a shift of T_m_ towards lower temperatures or towards destabilizing the protein. These compounds distort characteristics of the protein related to the protein maturation caused by PTMs, which are specifically the metal content and the disulfide bonds respectively [24,27,28] (Figure 5). From our results, it was obvious that the WT variant seemed to be more tolerant to PTM inhibition, since higher concentrations of the added compound (EDTA or DTT) were needed to achieve a significant T_m_ shift compared to SOD1(A4V) (Table S1).

### 2.2. Selection of the Chemical Library: LOPAC^®^

LOPAC^®^ (Library of Pharmacologically Active Compounds) was chosen to be screened for potent aggregation rescuers for the pathogenic ALS-related mutant SOD1(A4V). It is a commercially available library containing 1280 marketed drugs and well-characterized small molecules displaying lead-like or drug-like properties. Our choice was based on the fact that LOPAC is a widely used library containing compounds displaying multiple mechanisms of action, which target a large number and diverse set of biological receptors. In addition, it covers a broad range of molecular weights—from 36 to 1485 Da [84]. To our knowledge, this is the first time that a LOPAC^®^ library was used in the context of a random screening against ALS-related SOD1 variants for the discovery of inhibitors/rescuers of aggregation that are directly targeted to SOD1 (protein–drug interaction).

#### Optimizing the Conditions before Screening

**Figure 6:**
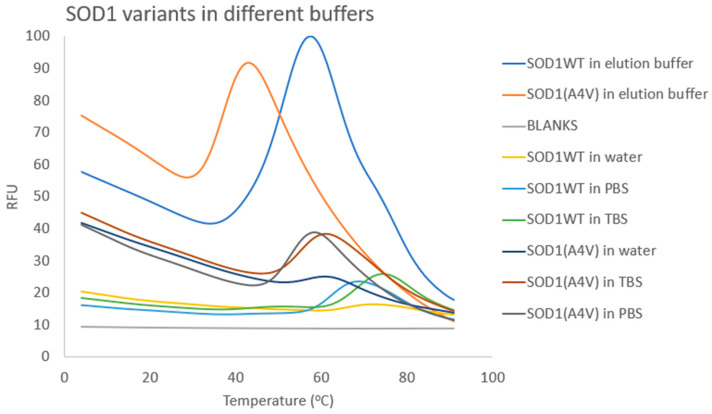
Examination of different buffers in regard to SOD1 stability. RFU corresponds to the normalized fluorescence for each compound from triplicate samples.

The desirable buffer for the chemical library screening should mimic physiological conditions; thus, Tris-buffered Saline (TBS) and phosphate-buffered saline (PBS) were used, along with water for comparison. Gel filtration chromatography (SEC) using these buffers was performed, and DSF spectra were extracted (Figure 6).

Based on the calculated T_m_ (Table S2), it is obvious that, when both SOD1 variants were resuspended in the two physiological buffers tested, a significant increase in T_m_ was observed, compared to the initial buffer tested (Table S2, Figure 6) (elution buffer, containing 250 mM imidazole and two-fold NaCl concentration compared to TBS and PBS) and water. TBS was chosen for the subsequent screening of the library because it presented a slightly larger increase in T_m_ compared to the PBS buffer, possibly implying that it was more appropriate for SOD1; nevertheless, the difference in T_m_ attributed to the two buffers was minimal for both SOD1 variants.

### 2.3. LOPAC^®^ Screening

**Figure 7:**
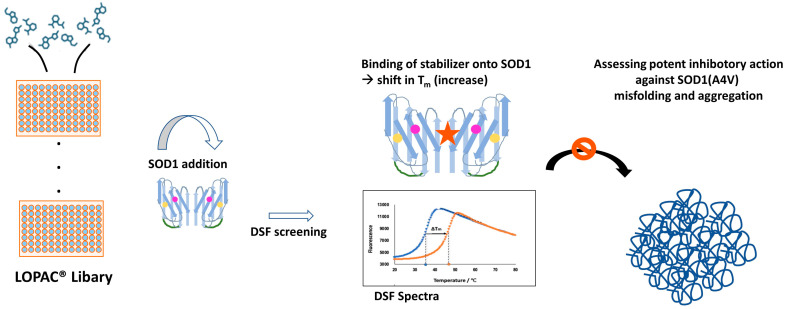
Workflow of LOPAC^®^ screening. The compounds are arranged (after proper dilution) in a 96-well plate format and the freshly purified SOD1(A4V) is added to the mixture. Following a short incubation period, DSF spectra are extracted. The compounds presenting a positive shift in the T_m_ of the mixture (denoted graphically with the red star onto the protein) will further be evaluated as potent aggregation inhibitors of SOD1 in kinetics experiments. The orange and pink dots represent Cu and Zn and the green round lines the disulfide bonds.

The main idea (Figure 7) was to incubate the library with SOD1(A4V) for an adequate amount of time to allow for interactions and to monitor for T_m_ shifts (if any). In general, higher T_m_ values correlate with a more stable protein [72,75]. If the candidate drug molecule added in the mixture binds specifically to the protein, it can stabilize the protein and cause an increase in Tm. An increase in Tm of 1–2 °C can be considered to be a sign of protein–drug interaction [79,85].

### 2.4. LOPAC^®^ Screening Results

**Figure 8:**
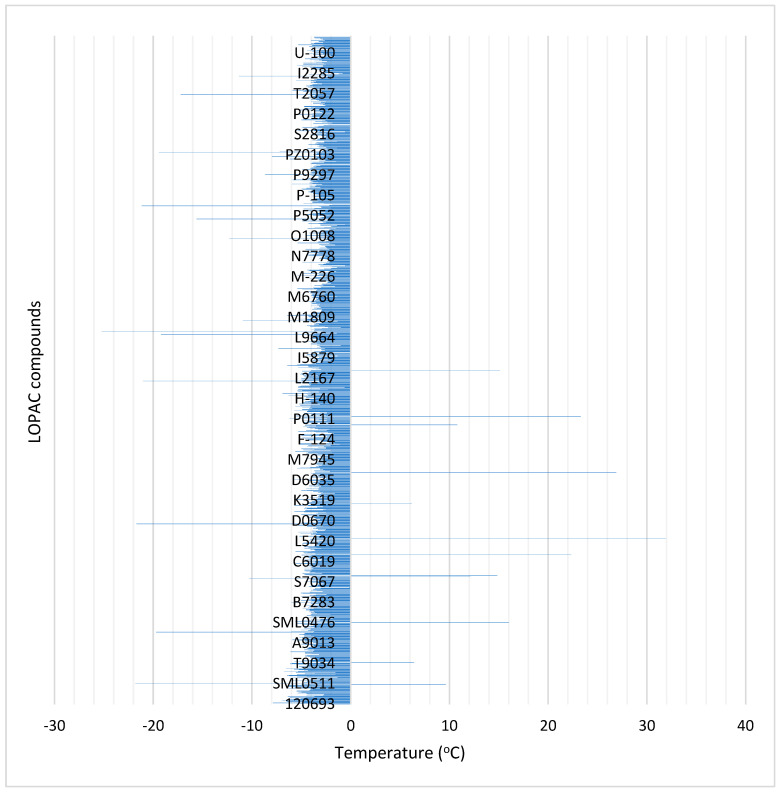
The difference in T_m_ values of SOD1(A4V) caused by the compounds of the LOPAC^®^ library. ΔT_m_ corresponds to the function: T_m_(SOD1(A4V)) − T_m_(SOD1(A4V) upon a compound’s presence).

The majority of the LOPAC^®^ compounds induced a difference in the T_m_ of the protein towards both directions: increasing and reducing it. All ΔT_m_ values are presented in the Supplementary Information Section (Table S3), and the differences in SOD1 T_m_ values caused by the presence of each compound is depicted in an overall chart in Figure 8.

Of the total number of 1280 LOPAC^®^ compounds (except for 12), 99.1% presented a negative ΔT_m_; in other words, they were able to decrease the T_m_ of the protein from 6 to 25 °C. Of the bulk of the compounds that presented a negative shift, 1260 of them, corresponding to almost 98% of the total number of the library’s chemicals, induced a negative shift in SOD1(A4V) T_m_ values, resulting in a difference from −7 °C to −0.5 °C. However, a 0.5 °C difference is small and generally considered negligible in the assessment of protein binders, whereas a difference of >1 °C is considered to indicate an interaction [79,85].

In order to select for target compounds to be further analyzed, some parameters should be established. For this selection, the readout was only the difference in T_m_ that each compound induces and not other features, e.g., the alteration in the fluorescence level of the curve [86,87]. Furthermore, several compounds induced a second shift, which was translated in the presence of a second T_m_ value upon their interaction with SOD1(A4V), and, interestingly, all cases shared the same pattern: the first shift was almost the same as the SOD1(A4V) protein was, while the second shift was located several degrees away, either to the left or the right. The second shift was clear and distinct, as it was characterized by a clear increase in fluorescence which subsequently dropped and exhibited a minimum of the first derivative of its curve, as a normal curve. The appearance of multiple transitions, generally, can be attributed to the different behaviors of protein domains, aggregation, or ligands stabilizing a portion of the protein [73].

#### Assessment of Compounds for Their Role as Potent Rescuers against SOD1(A4V) Misfolding and Aggregation

At first, we excluded the compounds that induced a negative difference in the T_m_ value, and our interest turned to the compounds able to cause only a positive difference in the T_m_ value of the protein. The compound Diacylglycerol Kinase Inhibitor II (D5794) was the only compound able to shift the T_m_ of the protein up to 6.2 °C while presenting a well-behaved DSF spectrum, e.g., one clear curve. The compounds cephalosporin C zinc salt (C3270), Cyclosporin A (C3662), Bosutinib (PZ0192), Rabeprazole sodium (SML0476), Bexarotene (SML0282), Ganciclovir (G2536), Calcimycin (C7522), Icaritin (SML0551), Theophylline (T1633), Aurothioglucose (A0606), and N,N-dihexyl-2-(4-fluorophenyl)indole-3-acetamide (D8555) exhibited a second positive shift with corresponding ΔT_m_ values ranging from 6 °C to 32 °C. In addition, the above-mentioned eleven compounds, apart from the second-highest T_m_, presented a T_m_ close to the T_m_ of the protein as is.

As far as negative shifts were concerned, the compounds Palmitoyl-DL-Carnitine chloride (P4509), Mifamurtide (SML0195), Olvanil (O0257), Idarubicin (I1656), Dihydrocapsaicin (M1022), Artemether (A9361), GNF-5 (SML0511), Fulvestrant (I4409), N-Oleoylethanolamine (O0383), TTA (T1698), AC-55649 (A9480), Carvedilol (C3993), CID11210285 hydrochloride (SML0698), and Ritanserin (R-103) presented ΔT_m_ values ranging from −10 °C to −26 °C, either as the only shift in the DSF spectrum—for the first six—or as a second shift, along with the shift close to the T_m_ value of the protein as is.

No matter how promising these large differences may seem, care should be taken in their interpretation. For this reason, the selection of the compounds was not only based on the ΔT_m_, but the DSF spectra were also taken into consideration. Since ΔT_m_ is calculated based on the overall minimum of the first derivative of the curve (fluorescence vs. temperature), the overview of the spectra was necessary, in order to avoid false positive hits deriving from artifacts or mathematic errors upon derivatization. In addition, during library screens, sometimes it is the aggregation between molecules of the compounds themselves that generate false results [88]. Although this problem is inevitable in most cases, it is resolved by undertaking further validation experiments.

### 2.5. Validation of the Action of Selected Compounds

**Figure 9:**
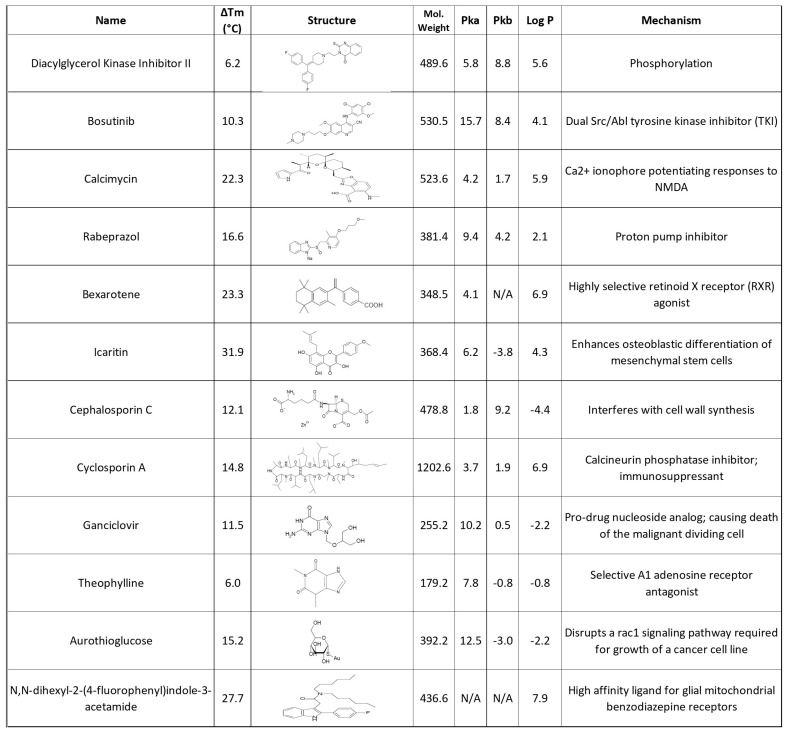
LOPAC^®^ compounds assessed as being able to cause a significant positive ΔT_m_ on SOD1(A4V) and as being worthy of further examination. In this table, some physicochemical PK properties and their mechanism of actions are also presented. (N/A represents not applicable measurement for the specific property).

**Figure 10:**
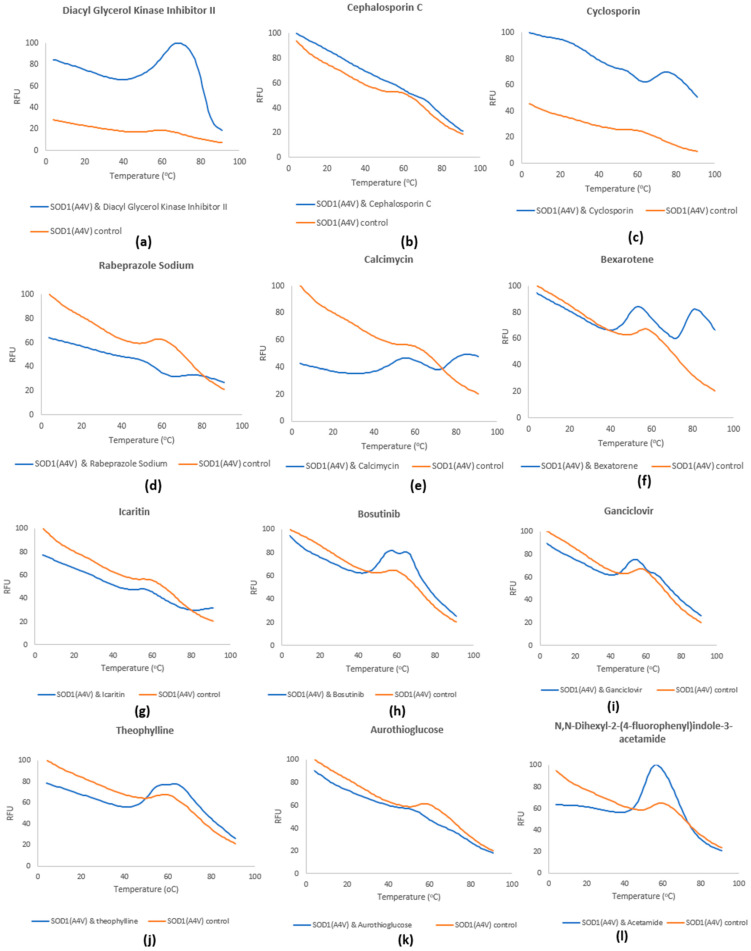
DSF spectra of the selected compounds. RFU corresponds to the normalized fluorescence for each compound from triplicate samples. The molecular ratio is SOD1(A4V):compound, 1:20 and these are (**a**) diakyl glycerol kinase inhibitor II, (**b**) cephalosporin C, (**c**) cyclosporin, (**d**) rabeprazole sodium, (**e**) calcimycin, (**f**) bexarotene, (**g**) icaritin, (**h**) bosutinib, (**i**) ganciclovir, (**j**) theophylline, (**k**) aurothioglucose, (**l**) N,N-dihexyl-2-(4-fluorophenyl)indole-3-acetamide.

Twelve compounds were selected (Figure 9) that caused T_m_ transitions that corresponded to positive shifts, as we were looking for compounds with a stabilizing action on the target protein, SOD1. These compounds were also selected because we aimed to cover both smaller and larger differences, allowing us to obtain the most representative samples of the compounds. The spectra of the selected compounds can be seen in Figure 10. This experiment verified that the spectra of these compounds with SOD1(A4V) were identical to those obtained during the screening of the initial library, suggesting a high level of reproducibility using this technique.

### 2.6. Identification of Binding Cites of the Selected Compounds Using Molecular Docking

The in vitro data suggest that SOD1(A4V) interacts with specific compounds from the LOPAC^®^ library. To further gain insight into the possible binding modes of these compounds in SOD1(A4V), in silico calculations were performed.

**Figure 11:**
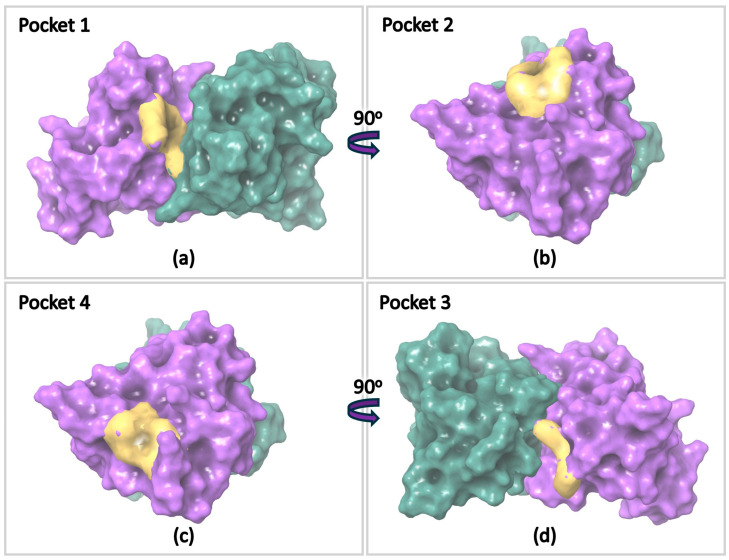
Docking pockets on the SOD1(A4V) surface are illustrated with yellow color. Green and purple colors denote the two monomers that create the dimer. Specifically, (**a**) Pocket 1 is located near Cys 11 residue, (**b**) Pocket 2 at the surface-exposed Trp32 site, (**d**) Pocket 3 is present close to the region where the intra-disulfide bond between the residues Cys57 and Cys146 is formed, and (**c**) Pocket 4 is positioned next to the electrostatic loop.

Four ligand-binding pockets have previously been identified in the dimer structure of SOD1, based on crystallographic data [89], and were used as references for the in silico calculations. One pocket is located near the dimer interface at the free Cys111 residue (Pocket 1), where covalent bonds can form with ligands such as Ebselen [20,21,37,90]. Additionally, non-covalent interactions have been characterized at sites including the surface-exposed Trp32 [91,92,93] (Pocket 2), the region near the intra-disulfide bond formed by residues Cys57 and Cys146 [89] (Pocket 3), and the site close to the electrostatic loop [89] (Pocket 4) (Figure 11).

Τhe aforementioned pockets were specified in the dimer of the SOD1 A4V crystallographic structure 6SPA, and Glide docking (Schrodinger Inc., New York, NY, USA), as well as induced-fit docking calculations (Schrodinger Inc.), was performed. Twelve compounds were selected based on the DSF results and docked in each pocket. The results are summarized in Table S6, where both docking and IFD scores indicate that the compounds bound predominantly at the dimer interface near Cys111 (Pocket 1). The lowest energy IFD poses are illustrated in Figures S4 and S5.

These theoretical models suggest that the binding of the experimentally defined molecules that interact with SOD1(A4V) is possible, preferably in a pocket that has been demonstrated to be important for the inhibition of aggregate formation [20,71].

## 3. Discussion

ALS is a biologically heterogeneous disorder in which genetics, environment, and aging all interrelate to generate the clinical phenotype [6,94]. A plethora of genes has been linked to the familial form of the disease, but most of the cases—characterized as sporadic—are still of unknown cause today. SOD1 is one of the most frequently mutated genes that account for ALS; this gene, historically, has been the most analyzed since its link with the familial form of the disease was discovered in 1993 [6,10]. SOD1, as a holo-enzyme, is highly thermostable and theoretically unlikely to undergo problematic folding [6,27]. However, mutations in SOD1 cause it to misfold and aggregate, leading to selective toxicity in motor neurons [6,24,37,41,86,95,96]. Despite extensive research, no common biophysical or biochemical mechanism for these mutations has been identified, and they result in significant phenotypic variability, even among patients with the same mutation.

Regarding the mutation we studied, A4V, it is not clear if it has an effect on the metal content of the protein: some published reports claim that this mutation is metalated in vivo [97], while others report that copper-loading specifically drops significantly due to this mutation [87,98]. We found that this mutation did not cause a significant reduction in enzyme activity. The A4V mutation confers distinct biochemical properties on SOD1: it leads to significant dimer instability, promoting its dissociation into monomers that aggregate more readily, compared to other mutations [24,31,37,93,99], thereby conferring strong gain of function characteristics [100,101].

Despite a plethora of compounds having been tested in the context of CTs (Tables S4 and S5), these compounds do not translate into effective therapies. The most recent example of such a compound is CuATSM, which failed to significantly alleviate neuronal pathology or astrogliosis in patients with ALS [102], underlying the need for expanded testing and innovative screening methods to overcome the limited success of previous efforts [103,104]. Consequently, drug discovery for ALS faces numerous challenges. Most importantly, ALS is heterogeneous, with various mutations and clinical presentations, suggesting there may not be a one-fits-all therapy. Furthermore, in the case of protein misfolding, unlike traditional drug targets with well-defined structures, misfolded oligomeric species and aggregates lack clarity and often exist in dynamically varying mixtures, a fact that complicates the pursuit of effective treatments even further. There are also many other inherent difficulties: firstly, animal models, e.g., SOD1 mice, while representing familial ALS (fALS), do not fully mimic sporadic ALS (sALS). In addition, the timing of therapy initiation is crucial, but predicting neurodegenerative disease onset is difficult. Drug effects may vary depending on this timing, leading to misinterpretations [80,81]. For instance, antioxidants like vitamin E delay symptom onset but do not affect survival, while riluzole slows the disease but not onset age [105]. Understanding the role of each mechanism, such as oxidative stress or glutamate toxicity, is key for drug selection [106]. Furthermore, designing trials is complicated by the need to optimize drug doses and bioavailability, considering differences between animal models and human patients [80,81]. Researchers are constantly trying to standardize ALS clinical trials by optimizing pharmacokinetics, considering genetic backgrounds, administering drugs pre- and post-symptom onset, publishing negative results, comparing different models [107], and—more recently—exploring biomarkers [108].

In our approach, we decided to employ a biophysical technique for the scanning of a chemical library, aiming to identify compounds that were able to stabilize the protein and alternate the mutant’s propensity to misfold and aggregate. This technique is DSF and is characterized by several advantages including simplicity, the low cost of the reagents, and its ability to perform medium- to high-throughput experiments, all of which are combined with extremely high sensitivity. In addition, we decided to include this application in the context of drug repurposing (also known as drug repositioning or therapeutic switching), since it is more advantageous in comparison to the classic drug discovery process due to the established safety profile of the drug, the shorter duration of the drug development phase, the reduced cost, and the limited risk of failure [56,109]. These features, combined with the concomitant cost reduction, create a major advantage, especially for ”orphan diseases” like ALS [56,109].

To our knowledge, there are no research data on the results of the in vitro interactions between LOPAC^®^ Library and SOD1. Despite the fact that all of these compounds are related to mechanisms completely different to oxidative protection, which is the mechanism of the target protein SOD1, it is interesting to study their direct interaction with SOD1 and discover if their potent role as stabilizers can be extended to, for example, acting as an inhibitor, modulator, or breaker of the SOD1 kinetic mechanism. Despite the fact that all of these compounds are related to mechanisms completely different to protein misfolding and aggregation, it is interesting to study their direct interaction with SOD1 and identify potent stabilizers in the context of random screening. In a next step, we can discover if their role as stabilizers can be extended to, for example, acting as an inhibitor, modulator, or breaker of the SOD1 kinetics mechanism.

Stabilizing the dimeric state of SOD1 holds therapeutic promise and many different approaches for dimer stabilization have been reported, including the following results that will be presented in the future: an engineered inter-subunit disulfide bond for the SOD1(A4V) variant that alleviated aggregation and restored enzymic activity [6,98,110,111], and a phosphomimetic mutation (T2-phosphorylation) able to thermodynamically stabilize the same variant, with the ability to inhibit the cytotoxic effect of the same destabilizing mutation (A4V) and increase cell viability [112]. Numerous other studies support the importance of SOD1 stabilizers, as each one binds onto different regions of the protein. The covalent attachment of small molecules to Cys111 is important: for example, maleimide linkers bridge the 9 Å gap across the SOD1 dimer interface via tethering Cys111 residues, increasing the thermal stability of the G93A and G85R mutations [113,114]; similarly, cyclic disulfides [115], mono-S-oxo derivatives [116], and cisplatin enhance the thermal stability of metal-free SOD1 and effectively prevent SOD1 aggregation within cells [117]. Ebselen and ebsulphur form covalent bonds with Cys111, functioning as bifunctional small molecule chaperones for various mutant forms of SOD1 [90]. Interestingly, a similar study using a DSF assay was used to investigate the stabilization effect of ebselen derivatives on SOD1(A4V), aiming for improved SOD1 dimer stabilization and drug-like properties; the selected compound demonstrated a significant delay in ALS disease onset in SOD1(G93A) mice and remarkable neuroprotective activities [20,118]. In addition, targeting the electrostatic loop in SOD1 is crucial for modulating its structure and function, as well as oligomer formation in ALS-related variants [119]. Small molecules, like the cyclic peptide HGG4FFQ, stabilize native SOD1 proteins and accelerate folding rates [120].

For the selection of the compounds, we chose to exclude those that decreased the T_m_ of the protein. This phenomenon can imply a non-specific binding or other mechanisms, such as a change in protein dynamics upon ligand binding [80]. Indeed, ligands can destabilize proteins by binding primarily to their unfolded state and thus reducing the protein T_m_ [121,122]. Nevertheless, the negative shift does not exclude interaction and binding to the native state [73]. Ligand binding to the unfolded protein state is a not well-understood process, since there are no data on the crystal structures of any unfolded proteins to help our understanding [121].

On the other hand, we chose compounds with the ability to increase the T_m_ of the protein, as this approach enabled more chances to discover a SOD1 stabilizer. The strategy of dimer stabilization holds promise as a therapeutic strategy against SOD1-linked ALS, with many different approaches published. Attempts to discover small molecules that bind to and stabilize the aberrant protein, thereby helping it to attain a native or near-native conformation and/or restoring its function, underline the importance of moving the equilibrium towards the stabilized form of the protein. The worthiness of this approach is reflected by the emergence of pharmacological chaperones as a relatively new therapeutic intervention against conformational diseases [62,64,65,123], including SOD1-linked ALS [71,115].

Concerning the interpretation of the differences in these proteins’ T_m_ values, it should be noted that a large shift in ΔT_m_ is not always correlated proportionally to the concentration or affinity of the ligands; a larger shift in T_m_ does not imply tighter binding [80,124,125,126]. Different ligands interacting with the same protein, bearing equivalent affinities, can result in different ΔT_m_ values. This phenomenon is related to the mechanism that each ligand uses to bind onto the protein; for instance, whether these interactions are more enthalpically or entropically driven. In the case of large shifts, this type of interaction often leads to more entropically dominated interactions [79].

Interestingly, within the LOPAC^®^ library, we identified compounds that have been associated with ALS disease in the past as possible disease modifiers. Some of them, such as Ceftriaxone (C5793) [127,128,129], Minocycline (M9511) [107,130] Gabapentin (G154) [131,132], Cyclosporin (C3662) [133], Verapamil (V4629) [134], Celecoxib (PZ0008) [135], Valproic acid (P4543) [136,137], Ropinirole (R2530) [138], and Thalidomide (T144) [139], have been tested in phase II/III clinical trials with inconclusive results or failure, but have shown promising results in ALS transgenic mice studies. Others, such as Pentoxiffyline (P1784) [140], Nimodipine (N149) [141], and Indinavir (SML0189) [142], have also been tested in clinical trials, resulting in non-significant improvements in patients or failure. However, no data on transgenic SOD1 animal models were found in the literature, meaning that the above compounds were chosen in the context of other mechanisms, like antioxidant or neuroprotective mechanisms. Finally, other compounds, such as Cisplatin (P4394) [117], Fluororacil (C1494) [93,143,144], and Isoproterenol (I2760) [93], have an inhibitory effect on SOD1 misfolding or a stabilizing effect, as found in in vitro studies.

Among the twelve selected compounds, Bexarotene has been tested in a SOD1(G93A) mouse model and significantly delayed motor function deterioration while extending mice survival up to almost 30%, by delaying early motor neuron degeneration and decreasing ubiquitylated inclusions [145]. Cyplosporin in a SOD1(G93A) mouse model demonstrated neuroprotective action when it crossed the blood–brain barrier and prolonged the survival of late-stage ALS transgenic animals, relating its mechanism of action to mitochondrial function [146]. Bosutinib, a Src/c-Abl inhibitor, boosted autophagy, reduced the amount of misfolded mutant SOD1 proteins, and attenuated the altered expression of mitochondrial genes, while also increasing the in vitro survival of ALS iPSC-derived motor neurons from patients with other forms of ALS, including sporadic ALS and other forms caused by mutations in other genes. Also, in a mouse model of SOD1(G93A), it slightly extended survival [147]. Interestingly, the same group proceeded to a phase I study of Bosutinib in patients with ALS, presenting some preliminary safety results [148]. Icaritin has been tested in a cell model with mutations in TDP-43, another gene implicated in ALS, and its protective effect, specifically the mitigation of mitochondrial damage and the reduction in oxidative stress, has been validated [149]. Lastly, Ganciclovir, when tested in a SOD1(G93A) mouse model, despite reducing reactive microglia and thereby alleviating another characteristic of ALS pathology, had no effect on motor neuron degeneration [150]. However, none of these compounds have ever been tested in the context of protein–drug interactions to examine their role as a potent chemical chaperone for SOD1.

Finally, the results from the molecular docking calculations indicate the compounds’ strong preference to bind at the dimer interface near the free Cys111 residue (Pocket 1) of the SOD1(A4V) variant, which aligns with known interactions observed in the SOD1WT protein. This observation suggests that the mutation in SOD1(A4V) may not significantly alter the ligand-binding dynamics at this key site, which is known to form covalent bonds with certain ligands, such as ebselen [20,37,90]. Additionally, the binding affinity at this pocket is further supported by the lowest energy poses observed in the IFD calculations. The consistent binding in this pocket across various compounds highlights its potential as a critical site for the therapeutic targeting of SOD1-related ALS.

## 4. Materials and Methods

### 4.1. Chemicals and Purification Columns

All chemical reagents (buffers, solutes, inducers, antibiotics, DTT, EDTA, culture media, and Coomassie Brilliant Blue R250) were purchased from Merck & Co., Inc. (Rahway, NJ, USA). The molecular marker/protein ladder used for SDS Polyacrylamide Gel Electrophoresis (PAGE) was the Bluestar Prestain from Nippon Genetics Co., Ltd., Bunkyo-ku, Tokyo, Japan. Sypro Orange Protein stain (5000× concentrate) in DMSO (Thermo Fischer Scientific Inc., Waltham, MA, USA) was diluted according to the needs of each experiment using the appropriate buffer or solution.

Propagated plasmids were purified using the NucleoSpin^®^ Plasmid from Macherey-Nagel (Düren, Nordrhein-Westfalen, Germany). For recombinant human-SOD1 purification from bacterial cells, Ni-NTA Agarose resin from Qiagen (Venlo, The Netherlands) was used.

### 4.2. E. coli Cultures and hSOD1 Recombinant Production

*E. coli* cells BL21 trxB (DE3) from Merck & Co., Inc. (Rahway, NJ, USA), freshly transformed with the expression vector pASK75-flag-SOD1-6His (constructed as described [86] and kindly gifted from the authors of the same study), were used for protein production. Single colonies grown in Luria–Bertani (LB) agar plates with the appropriate antibiotic were picked and used to inoculate 6 mL of liquid LB broth overnight. Cells from overnight cultures diluted to OD600 0.3–0.5 were used to inoculate cultures of 250 mL or 500 mL LB media containing 100 μg/mL ampicillin (pASK75 expression vector) and 50 μg/mL kanamycin (BL21 trxB cells’ resistance) as well as 200 μΜ CuCl_2_ and 200 μΜ ZnSO_4_ (salts carrying the metals/co-factors of SOD1). Cultures were grown at 37 °C with adequate aeration in a shaking incubator, and, when OD600 reached 0.7–0.8, SOD1 production was induced by the addition of ATC (Anhydroteracycline) at a final concentration of 200 ng/mL [151]. The stock solution of ATC was prepared at a concentration of 2 mg/mL, dissolved in pure ethanol, protected from light, and stored at −20 °C. The working solution of ATC was further diluted to 0.02 mg/mL in ethanol and was added to the cultures, diluted to a ratio of 1/100, in reference to the culture volume. All solutions were filtered using 0.2 μm syringe filters (Corning, Tewksbury, MA, USA) and transferred to sterilized vials. The temperature was switched to 19 °C for an overnight induction lasting about 19 h.

### 4.3. SOD1 Purification

Cell pellets were obtained from 250 mL of SOD1 overexpression cultures collected by centrifugation at 6000 rpm for 15 min at 4 °C (Juan KR25i, DJB CareLab, Newport Pagnell, UK). The cell pellets were washed once with 20 mL lysis buffer and centrifuged again under the same conditions. The resulting pellets were resuspended in 19 mL lysis buffer (50 mM NaH_2_PO_4_, 300 mM NaCl, and 10 mM imidazole; pH 8.0) and lysed through sonication in ice (Vibra Cell^TM^, Sonics & Materials Inc., Newtown, CT, USA). The soluble cell lysate was collected by means of centrifugation (FastGene HighSpeed Mini Centrifuge, Nippon Genetics Co., Ltd., Bunkyo-ku, Tokyo, Japan) at 13,000 rpm for 25 min at 4 °C, loaded onto 1 mL of Ni-NTA agarose resin (Qiagen, Venlo, The Netherlands), and equilibrated with a lysis buffer (50 mM NaH_2_PO_4_, 300 mM NaCl, and 10 mM imidazole; pH 8.0). The column was washed with wash buffer (50 mM NaH_2_PO_4_, 300 mM NaCl, and 20 mM imidazole; pH 8.0), and SOD1 was eluted from the column using elution buffer (50 mM NaH_2_PO_4_, 300 mM NaCl, and 250 mM imidazole; pH 8.0). SOD1 was further purified using size-exclusion chromatography (SEC), using a custom-made gel filtration column (id 1.2 × 100 cm) packed with Sephacryl^TM^ S-200 (GE Healthcare, Chicago, IL, USA) in Tris-buffered saline (TBS), pH 7.4. The chromatographic system comprised an ISMATEC pump (Thermo Fischer Scientific Inc., Waltham, MA, USA) and a UV detector, namely Pharmacia LKB-Uvicord SII (American Laboratory Trading, East Lyme, CT, USA). The SOD1 concentration was estimated using UV spectrophotometry (Shimadzu, Nakagyo-ku, Kyoto, Japan) at 280 nm [28].

### 4.4. Polyacrylamide Gel Electrophoresis (PAGE)

For denaturing SDS-PAGE, the samples were boiled for 5 min and diluted in a loading solution (Laemli buffer) containing SDS and mercaptoethanol. Of the sample, 20 μL was loaded onto a 15% gel. Gels ran at 30 mA inside the stacking gel area and at 70 mA until the end (when the dye was about to leave the gel).

For native-PAGE, the protein samples were diluted with the loading solution (Laemli buffer) that did not contain any denaturing agents. All processes were carried out on ice and the samples were not boiled. Of each sample, 10 or 20 μL was loaded onto SDS-free 10% gels. Gels ran at RT, using a running buffer that did not contain denaturing agent. The samples ran using a constant current at 30 mA, until the front of the dye just came out of the gel.

Protein staining was undertaken using Coomassie Brilliant Blue R250. After protein electrophoresis, the gels were immersed and incubated for about 17–20 min in RT in Coomassie solution. The ingredients of 200 mL of this solution were as follows: 90 mL water, 20 mL acetic acid, 90 mL methanol, and 0.50 g of Coomassie Brilliant Blue R250 Powder.

### 4.5. In-Gel Activity Measurement

SOD1 enzymatic activity was monitored using the Riboflavin/Nitroblue Tetrazolium (NBT) gel-based assay [152]. The principle of this method involves two combined reactions. Firstly, riboflavin produces free radicals when exposed to UV or visible light (420–560 nm), because a photon activates this molecule, leading to its oxidation in the presence of an electron donor [153,154,155]. Secondly, the reduction of NBT increases its absorbance at 560 nm and produces a blue-colored formazan. SOD1 competes with NBT for O2-, appearing as an achromatic zone on Native-PAGE by inhibiting the blue precipitate formation [152,155,156].

Ten μL of SOD1 (WT or mutant) at a of concentration of 30–40 μm—after being diluted with Laemli buffer (that did not contain SDS or a reducing agent)—was analyzed in a 10% polyacrylamide gel. Following electrophoresis, the gel was washed thrice with dH_2_O. The gel was stained using 12.5 mL of a solution that contained 50 mM sodium phosphate buffer (pH 8.0), 66 mg riboflavin, 4 mg NBT, and 50 μL TEMED, after incubation for 1 h at 4 °C in the dark while shaking. After exposure to light, SOD1 activity could be seen as colorless bands on the gel, because SOD1 scavenges free radicals, inhibiting the reduction of NBT, which causes the blue color through the formation of the insoluble formazan.

In order to determine how the A4V mutation alters SOD1 activity, we analyzed the bands of the previous in-gel activity measurements using the ImageJ application (Version 1.54j, 12 June 2024). This is a public domain Java image-processing program for color images. Each image was first subjected to grayscale conversion of 32-bit. The image brightness was adjusted accordingly in order to increase the signal-to-noise ratio. The band to be quantified from each line of the gel was indicated by the rectangle tool and was marked for subsequent analysis using the following command: “Analyze” menu > “Gels” > “Select first lane”. The next bands were marked for analysis using the following command: “Analyze” menu > “Gels” > “Select next lane”. The graphical depiction of the band intensity was achieved using the command “Plot lanes” in the “Analyze” menu, and the area under the peak from each band was marked. Finally, using the “Wand” tool, the window with the results opened, containing all the values from the areas under each band peak selected, for further calculations.

### 4.6. Differential Scanning Fluorimetry (DSF) Measurements

DSF was carried out using a Bio-Rad CFX Real-Time PCR machine (Bio-Rad Laboratories, Inc., Hercules, CA, USA). Proteins, at 80 μM for SOD1 WT and 20 μM for the A4V mutation, with or without the selected compounds, were mixed, incubated for about 15 min under mild shaking (orbital shaker Kisker Biotech GmbH & Co, Steinfurt, Germany), and were measured thereafter. The final concentration of DMSO in the mixture was 0.02% (*v*/*v*) for the samples during LOPAC^®^ library screening. SYPRO Orange dye, at a 10× concentration, was used as the florescent probe. Sypro Orange dye (the excitation and emission wavelengths of which were 492 and 610 nm, respectively [124]) was chosen due to its high signal-to-noise ratio [75].

For LOPAC^®^ screening, the compounds were diluted 20 times in TBS buffer from the stock solution (10 mM in 10% DMSO) to a final concentration of 500 μM. The freshly purified SOD1(A4V) dimeric protein was then added at a concentration of 20 μM (1/25 ratio). The above initial master mix of the SOD1 protein with SYPRO Orange stain was added to each rack containing the compounds of the library and was mildly pipetted to ensure adequate mixing. A mild 20-min incubation on a shaking platform, under RT conditions (orbital shaker from Kisker Biotech GmbH & Co, Steinfurt, Germany) followed, in the absence of light. Each compound was tested in triplicate. A temperature scan was performed from 4 °C to 91 °C at a ramp rate of 2 °C/min. The analysis of melting temperature (T_m_) was calculated from the minimum of the first derivative of the RFU curve extracted from the PCR apparatus. The data were analyzed using the following online tool: https://gestwickilab.shinyapps.io/dsfworld/ (accessed on 14 June 2024) [157].

### 4.7. Statistical Analysis

All experiments were repeated in triplicate. Microsoft Office 2011 (Microsoft Corporation, Redmond, WA, USA) was used to analyze the experimental data.

### 4.8. Molecular Docking Calculations

The crystal structure of SOD1 A4V, with the PDB ID 6SPA [20], was selected for in silico calculations, focusing on chains A and C, which form a dimer. The system was prepared and minimized using the “Protein Preparation Wizard” tool within Maestro (Schrödinger Inc., New York, NY, USA) [158]. Ligand preparation was conducted using LigPrep (Schrödinger Inc.) [159] for the 11 ligands, while the “Minimization” tool in the Macromodel (Schrödinger Inc.) [160] was used specifically for Cyclosporin. The alignment of crystal structures 7XX3 [89] and 6SPJ [20] to 6SPA was carried out using the “Protein Structure Alignment” tool (Schrödinger Inc.) [161]. Naphthalene-catechol and ebselen derivative 1, co-crystallized with SOD1 and SOD1 A4V in 7XX3 and 6SPJ, respectively, were used as reference points to specify the binding pockets of SOD1(A4V). The “Receptor Grid Generation” tool within Glide (Schrödinger Inc.) [162] was employed to create grids for each of the four binding pockets of SOD1, using default parameters and Phialomustin-B, napthalene-catechol, and Ebselen derivative 1, as the centroids for the grid boxes. Separate grids were generated for Cyclosporin, with a ligand diameter midpoint box length of approximately 40 Å along the X, Y, and Z axes, to accommodate docked ligands up to approximately 36 Å in length. Docking calculations for each of the eight grids were executed using Glide (Schrödinger Inc.) [162] under default settings. Additionally, induced fit docking (IFD) calculations were performed, following the standard protocol of the IFD tool (Schrödinger Inc.) [163].

The initial Glide docking process was employed, allowing flexible scaling of the Van der Waals (VdW) radii and the generation of a maximum of 20 poses per ligand. Subsequently, Prime was utilized for side-chain prediction and minimization for each protein–ligand complex. Complexes with energies near the lowest-energy structure were subjected to redocking with Glide, and the IFD score was calculated to estimate binding energy.

## 5. Conclusions

Despite SOD1 being one of the most thermostable proteins and thus theoretically less susceptible to problematic folding, mutations such as A4V induce a tendency for misfolding and aggregation. This aberrant behavior leads to selective toxicity in MNs. ALS has also been associated with various other genes, contributing to the familial form of the disease (fALS). Additionally, sporadic cases of ALS (sALS) further highlight the complexity and variability in the clinical manifestations of the disorder. We proposed an easy and fast biophysical screening for the identification of SOD1 stabilizers from a chemical library (LOPAC^®^), and we managed to identify twelve compounds with potential stabilizing action. Furthermore, our in silico studies and molecular docking results support our hypothesis and highlight that compounds binding near the free Cys111 residue (Pocket 1) are promising candidates for stabilizing the SOD1 dimer and potentially modulating the disease’s progression. This pocket remains a critical therapeutic target, due to its involvement in covalent interactions with stabilizing ligands.

The urgent need for novel and effective therapies for ALS is underscored by its significant socio-economic burden and projected increase in patient numbers globally. While in vitro studies offer valuable insights into protein–ligand interactions, they also present limitations by simulating an artificial environment that may not fully capture the complexities of cellular conditions. Nevertheless, these studies serve as crucial preliminary steps in identifying compounds with potential therapeutic benefits that could be translated in developing effective treatments.

## Data Availability

All data obtained throughout this study are incorporated within the manuscript and the Supplementary Materials.

## Supplementary Materials

The following supporting information can be downloaded at https://www.mdpi.com/article/10.3390/ph17101286/s1, Figure S1: The positions of most known fALS-associated mutations are listed vertically in red letters below the naturally occurring aminoacids in black. The secondary structure elements are mentioned above the primary sequence; Figure S2: pASK75SOD1WT harvested from 0.25 L culture of BL21 trxB cells, incubated at 19 °C after o/n induction; Figure S3: pASK75SOD1(A4V) harvested from 0.25 L culture of BL21 trxB cells, incubated at 19 °C after o/n induction; Figure S4: The lowest energy induced Fit docking poses of Aurothioglucose (a), Bexarotene (b), Bosutinib (c), Calcimycin (d) and Cephalosporin (e) in Pocket 1, near to the Cys111 residue are presented. Figure (f) shows the Glide docking result of Cyclosporin in the same site; Figure S5: The lowest energy induced Fit docking poses of Diacylglycerol kinase inhibitor II (a), Icaritin (c), N,N-Dihexyl-2-(4-fluorophenyl)indole-3-acetamide (d) Rabeprazole (e) and Theophylline (f) in Pocket 1, near to the Cys111 residue are illustrated. Figure (b) shows the IFD result of Ganciclovir in Pocket 3 near the intra-disulfide bond of Cys57 and Cys146; Table S1: Calculated T_m_ after the addition of compounds that disrupt the PTM maturation of SOD1: EDTA, a chelator and DDT, a reducing agent; Table S2: Calculated T_m_ following SEC of both SOD1 variants using different buffers; Table S3: Table summarizing all the compounds from LOPAC^®^ library and the difference in T_m_ of SOD1(A4V) they provoked; Table S4: Compounds tested in previous clinical trials of Phase II or III in ALS patients with inconclusive results or failure to demonstrate significantly importance or implications with safety. Including those by efficacy in studies from SOD1 murine models; Table S5: Recent compounds (or approaches) with current interest and on going trials 48. They do not show direct connection with SOD1 aggregation but were firstly used in SOD1-associated ALS animal models; Table S6: The results from both Rigid and Induced Fit Docking are presented. References [8,46,56,105,107,127,128,129,130,131,132,133,134,135,136,137,138,139,140,141,142,146,147,148,164,165,166,167,168,169,170,171,172,173,174,175,176,177,178,179,180,181,182,183,184,185,186,187,188,189,190,191,192,193,194,195,196,197,198,199,200,201,202,203,204,205,206,207,208,209,210,211,212,213,214,215,216,217,218,219,220,221,222] are cited in the Supplementary Materials.
