# Supplementary material for: Discovery of Novel Inhibitors against ALS-Related SOD1(A4V) Aggregation through the Screening of a Chemical Library Using Differential Scanning Fluorimetry (DSF)
Source: Pharmaceuticals (Basel). 2024 Sep 27;17(10):1286. doi: 10.3390/ph17101286 (PMC11510448; doi:10.3390/ph17101286)
Supplement: Supplementary file 1 [file pharmaceuticals-17-01286-s001.zip › pharmaceuticals-3084552-supplementary.pdf]

Discovery of Novel Inhibitors against ALS-Related SOD1(A4V) Aggregation through the Screening of a Chemical Library Using Differential Scanning Fluorimetry (DSF)

Maria Giannakou [1,2], Ifigeneia Akrani [3], Angeliki Tsoka [1], Vassilios Myrianthopoulos [3], Emmanuel Mikros [3], Constantinos Vorgias [1] and Dimitris G. Hatzinikolaou [2,*]

[1] Biochemistry and Molecular Biology Unit, Department of Biology, National and Kapodistrian University of Athens, 15784 Zografou, Greece
[2] Enzyme and Microbial Biotechnology Unit, Department of Biology, National and Kapodistrian University of Athens, 15784 Zografou, Greece
[3] Laboratory of Pharmaceutical Chemistry, Department of Pharmacy, National and Kapodistrian University of Athens, 15784 Zografou, Greece; iakrani@pharm.uoa.gr (I.A.)
* Correspondence: dhatzini@biol.uoa.gr

Supplementary Figures

Figure S1: The positions of most known fALS-associated mutations are listed vertically in red letters below the naturally occurring aminoacids in black. The secondary structure elements are mentioned above the primary sequence. In each monomer, the β-strands are shown in light green, and all the loops are also pointed out including functional loops IV and VII. In this scheme, the disulfide bond between Cys57-Cys146 is indicated by blue, the non-conserved free Cys (Cys6 and Cys111) are shown in orange, while the metals are presented as spheres: copper in magenta and zinc in yellow, respectively. The mutations depicted here were reviewed from /and the structural information for SOD1 are based on [1,2]. 3
Figure S2: pASK75SOD1WT harvested from 0.25L culture of BL21 trxB cells, incubated at 19°C after o/n induction. Samples loaded: (1) Molecular Weight Marker, (2) before induction, (3) after induction total cell lysate, (4) soluble cell lysate, (5) flowthrough plus wash buffer off the column, (6) dead volume 500μL, (7) 1st elution 500uL (18 mg/mL), (8) 2nd elution 1000μL, (9) 3rd elution 500μL, (10) pellet after lysis-insoluble fraction. 4
Figure S3: pASK75SOD1(A4V) harvested from 0.25L culture of BL21 trxB cells, incubated at 19°C after o/n induction. Samples loaded: (1) Molecular Weight Marker, (2) before induction, (3) after induction total cell lysate, (3) soluble cell lysate, (4) flowthrough plus wash buffer off the column, (5) dead volume 500μL, (6) 1st elution 500μL (14 mg/ml), (7) 2nd elution 1000μL, (8) 3rd elution 500μL, (9) pellet after lysis-insoluble fraction. 4
Figure S4: The lowest energy induced Fit docking poses of Aurothioglucose (A), Bexarotene (B), Bosutinib (C), Calcimycin (D) and Cephalosporin (E) in Pocket 1, near to the Cys111 residue are presented. Figure (F) shows the Glide docking result of Cyclosporin in the same site. 5
Figure S5: The lowest energy induced Fit docking poses of Diacylglycerol kinase inhibitor II (A), Icaritin (C), N,N-Dihexyl-2-(4-fluorophenyl)indole-3-acetamide (D) Rabeprazole (E) and Theophylline (F) in Pocket 1, near to the Cys111 residue are illustrated. Figure (B) shows the IFD result of Ganciclovir in Pocket 3 near the intra-disulfide bond of Cys57 and Cys146. 6

Supplementary Tables

Table S1: Calculated T_m after the addition of compounds that disrupt the PTM maturation of SOD1: EDTA, a chelator and DDT, a reducing agent. Their effect has a dose-dependant effect on SOD1 variants. 7
Table S2: Calculated T_m following SEC of both SOD1 variants using different buffers. 7
Table S3: Table summarizing all the compounds from LOPAC® library and the difference in T_m of SOD1(A4V) they provoked. Compounds are presented by their catalogue number and the ΔT_m corresponds to the function: T_m(SOD1(A4V))-T_m(SOD1(A4V) upon compound's presence). SD is not displayed in the table and it was ranging from 0-0.4°C. Compounds with (*) presented a second higher T_m, and compounds with (#) presented a second lower T_m........ 8
Table S4: Compounds tested in previous clinical trials of Phase II or III in ALS patients with inconclusive results or failure to demonstrate significantly importance or implications with safety. Including those by efficacy in studies from SOD1 murine models. (Those carried out before 1993 are not related to SOD1)........ 17
Table S5: Recent compounds (or approaches) with current interest and on going trials [48]. They do not show direct connection with SOD1 aggregation but were firstly used in SOD1-associated ALS animal models........ 20
Table S6: The results from both Rigid and Induced Fit Docking (IFD) are presented. The docking poses with the lowest energy for each ligand, as calculated using Schrödinger Maestro's Glide and IFD software functions, are shown in the table. 21

Supplementary References

References........ 22

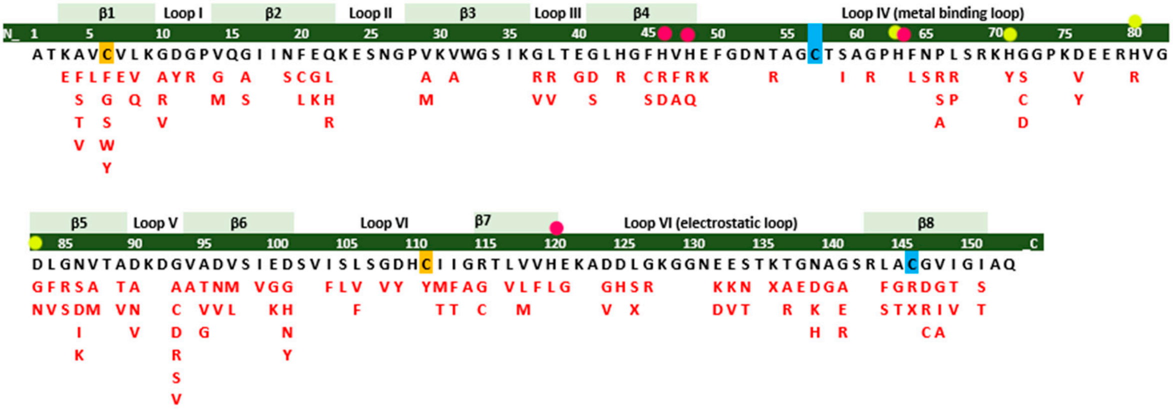

Figure S1: The positions of most known fALS-associated mutations are listed vertically in red letters below the naturally occurring aminoacids in black. The secondary structure elements are mentioned above the primary sequence. In each monomer, the β-strands are shown in light green, and all the loops are also pointed out including functional loops IV and VII. In this scheme, the disulfide bond between Cys57-Cys146 is indicated by blue, the non-conserved free Cys (Cys6 and Cys111) are shown in orange, while the metals are presented as spheres: copper in magenta and zinc in yellow, respectively. The mutations depicted here were reviewed from /and the structural information for SOD1 are based on [1,2].

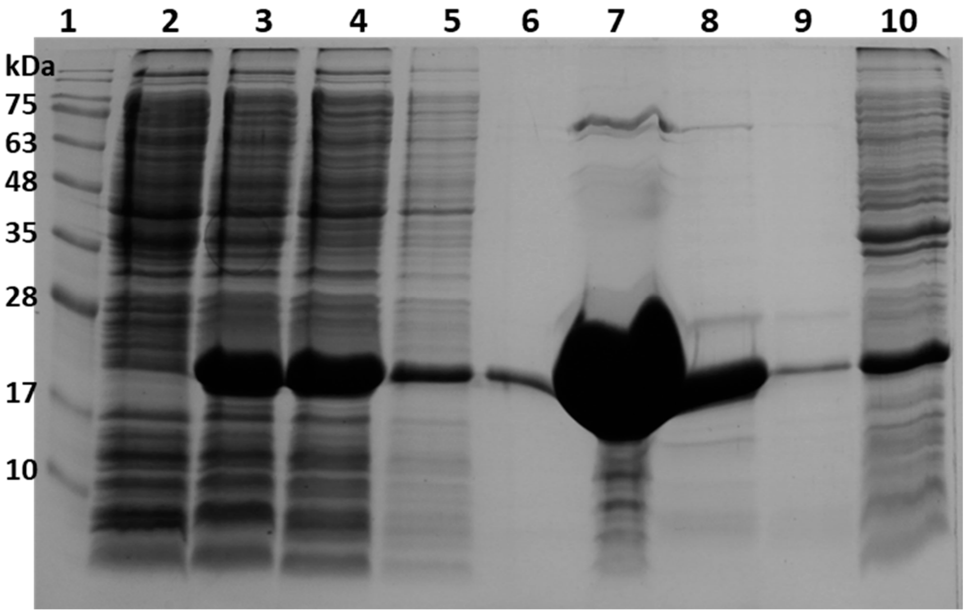

Figure S2: pASK75SOD1WT harvested from 0.25L culture of BL21 trxB cells, incubated at 19°C after o/n induction. Samples loaded: (1) Molecular Weight Marker, (2) before induction, (3) after induction total cell lysate, (4) soluble cell lysate, (5) flowthrough plus wash buffer off the column, (6) dead volume 500µL, (7) 1st elution 500uL (18 mg/mL), (8) 2nd elution 1000µL, (9) 3rd elution 500µL, (10) pellet after lysis-insoluble fraction.

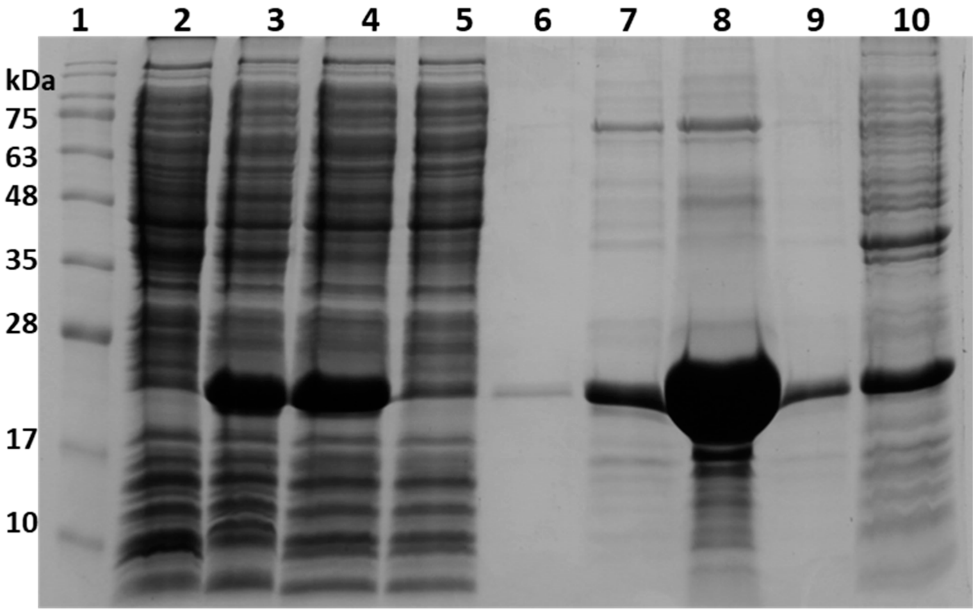

Figure S3: pASK75SOD1(A4V) harvested from 0.25L culture of BL21 trxB cells, incubated at 19°C after o/n induction. Samples loaded: (1) Molecular Weight Marker, (2) before induction, (3) after induction total cell lysate, (3) soluble cell lysate, (4) flowthrough plus wash buffer off the column, (5) dead volume 500µL, (6) 1st elution 500µL (14 mg/ml), (7) 2nd elution 1000µL, (8) 3rd elution 500µL, (9) pellet after lysis-insoluble fraction.

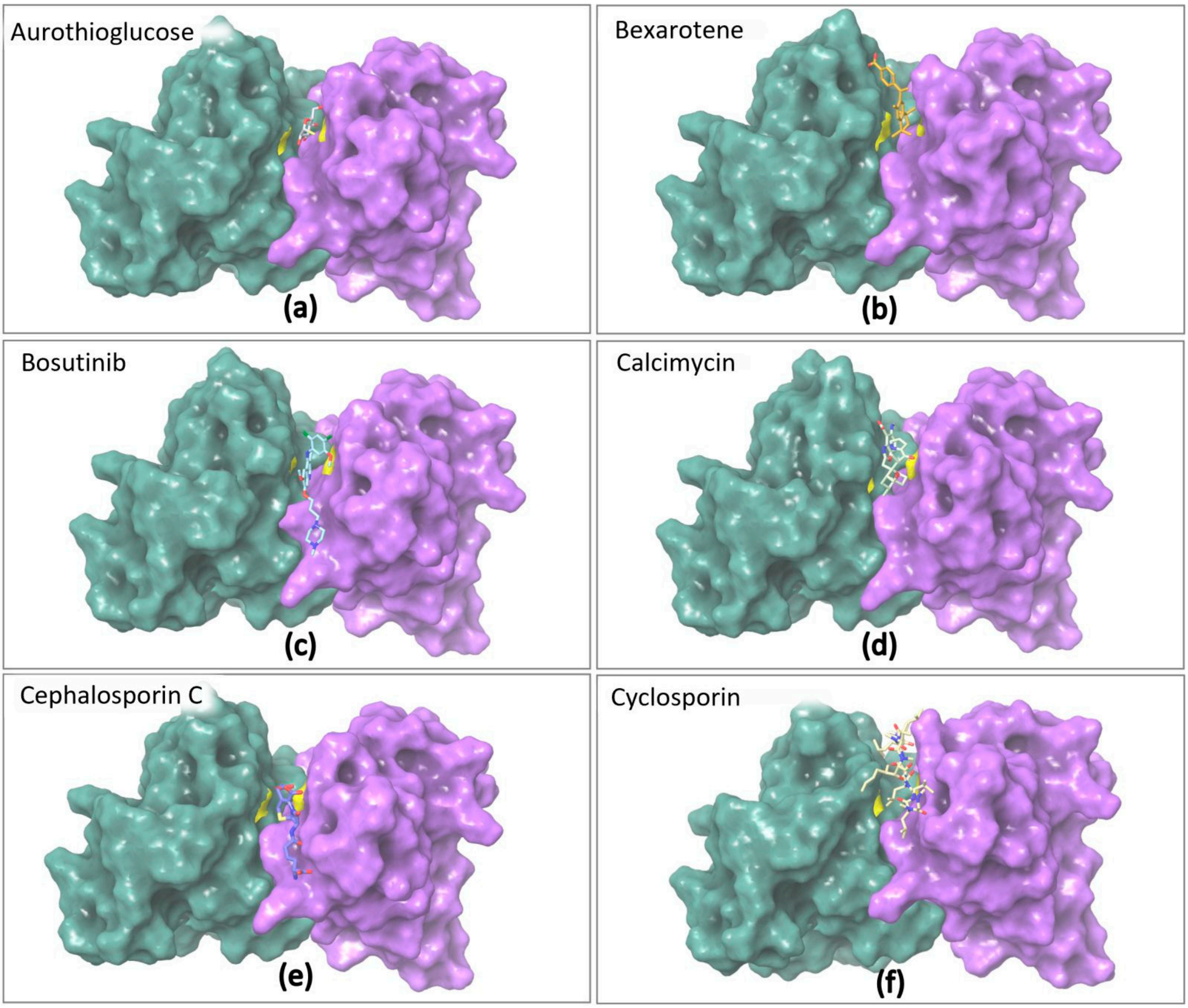

Figure S4: The lowest energy induced Fit docking poses of (a) Aurothioglucose, (b) Bexarotene, (c) Bosutinib, (d) Calcimycin and (e) Cephalosporin in Pocket 1, near to the Cys111 residue are presented. Figure (f) shows the Glide docking result of Cyclosporin in the same site.

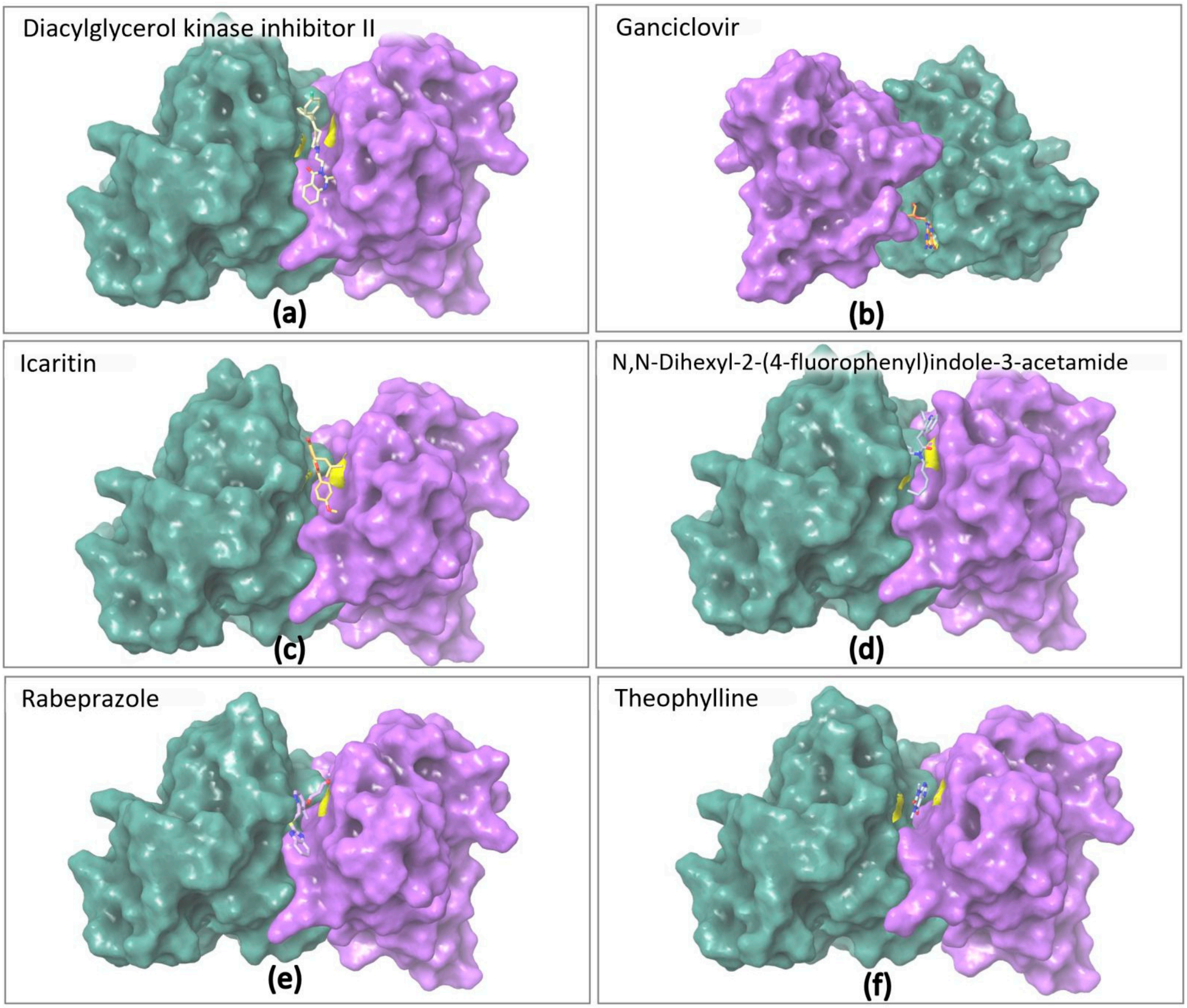

Figure S5: The lowest energy induced Fit docking poses of (a) Diacylglycerol kinase inhibitor II, (b) Ganciclovir, (c) Icaritin, (d) N,N-Dihexyl-2-(4-fluorophenyl)indole-3-acetamide, € Rabeprazole and (f) Theophylline in Pocket 1, near to the Cys111 residue are illustrated. Figure (B) shows the IFD result of Ganciclovir in Pocket 3 near the intra-disulfide bond of Cys57 and Cys146.

Table S1: Calculated T_m after the addition of compounds that disrupt the PTM maturation of SOD1: EDTA, a chelator and DDT, a reducing agent. Their effect has a dose-dependant effect on SOD1 variants.

**EDTA addition**			**DTT addition**		
**SOD1WT**	**T_m (ºC)**	**SD**	**SOD1WT**	**T_m (ºC)**	**SD**
SOD1WT as is	50.4	0	SOD1 WT as is	50.7	0.14
0.015mM	49.4	0.07	1mM	47.6	0
0.03mM	47.0	0.85	5mM	36.4	0.14
0.1mM	42.8	0.07	10mM	35.4	0.64
0.5mM	42.7	0.07	30mM	N/A	
1mM	42.3	0.07	60mM	N/A	
2mM	42.4	0	**SOD1(A4V)**	**T_m (ºC)**	**SD**
5mM	42.3	0	SOD1 A4V as is	35.8	0.07
**SOD1(A4V)**	**T_m (ºC)**	**SD**	0.025mM	35.5	0.07
SOD1(A4V) as is	35.6	0.14	0.2mM	35.3	0.07
0.08mM	35.3	0.14	1mM	32.7	0.07
0.15mM	34.8	0.07	5mM	N/A	
0.5mM	31.5	0.07	10mM	N/A	
1mM	31.5	0			

Table S2: Calculated T_m following SEC of both SOD1 variants using different buffers.

Condition	T_m (ºC)	SD
**SOD1(A4V) in water**	56.2	0.5
**SOD1(A4V) in PBS**	52.3	0.1
**SOD1(A4V) in TBS**	54.7	0.2
**SOD1(A4V) in elut. buf**	36.1	0.1
**SOD1WT in water**	66.1	0.1
**SOD1WT in PBS**	62.1	0
**SOD1WT in TBS**	67.9	0.1
**SOD1WT in elut. buf**	50.4	0

Table S3: Table summarizing all the compounds from LOPAC® library and the difference in T_m of SOD1(A4V) they provoked. Compounds are presented by their catalogue number and the ΔT_m corresponds to the function: T_m(SOD1(A4V))-T_m(SOD1(A4V) upon compound's presence). SD is not displayed in the table and it was ranging from 0-0.4°C. Compounds with (*) presented a second higher T_m, and compounds with (#) presented a second lower T_m.

Cat No	ΔT_m	Cat No	ΔT_m	Cat No	ΔT_m	Cat No	ΔT_m	Cat No	ΔT_m	Cat No	ΔT_m	Cat No	ΔT_m
**SML0195 (#)**	-3.8, -25.2	**SML0666**	-4.7	**T2265**	-4.1	**S0752**	-3.8	**R2751**	-3.4	**A2731**	-2.9	**S7198**	-2.2
**C3993 (#)**	-4.1, -21.7	**C1335**	-4.7	**T5515**	-4.1	**S7771**	-3.8	**R5010**	-3.4	**B1686**	-2.9	**SML0744**	-2.2
**SML0698 (#)**	-3.4, -21.2	**C9847**	-4.7	**T7508**	-4.1	**SML0597**	-3.8	**S0278**	-3.4	**C-106**	-2.9	**S2250**	-2.2
**I1656**	-21.0	**D3648**	-4.7	**U-100**	-4.1	**B2515**	-3.7	**S4063**	-3.4	**D7802**	-2.9	**B5002**	-2.1
**O0257**	-19.2	**D4434**	-4.7	**SML0584**	-4.1	**B4558**	-3.7	**S7395**	-3.4	**E1279**	-2.9	**D9071**	-2.1
**O0383 (#)**	-3.9, -12.3	**H2138**	-4.7	**A2251**	-4.1	**C0768**	-3.7	**S8010**	-3.4	**I6138**	-2.9	**F4381**	-2.1
**M1022**	-10.9	**I0782**	-4.7	**A2385**	-4.1	**C2505**	-3.7	**B7148**	-3.3	**K1003**	-2.9	**U-116**	-2.1
**A9361**	-10.3	**M1195**	-4.7	**B5556**	-4.1	**A-140**	-3.7	**P5749**	-3.3	**K1888**	-2.9	**B-173**	-2.0
**P9375**	-8.7	**C3909**	-4.7	**B9061**	-4.1	**A7275**	-3.7	**PZ0129**	-3.3	**L-134**	-2.9	**E-114**	-2.0
**O2139**	-8.0	**C4418**	-4.7	**C4382**	-4.1	**A7655**	-3.7	**PZ0172**	-3.3	**M3778**	-2.9	**E7138**	-2.0
**190047**	-7.9	**C4520**	-4.7	**C5040**	-4.1	**A8423**	-3.7	**SML0530**	-3.3	**M4008**	-2.9	**F3764**	-2.0
**K2628**	-7.3	**C4662**	-4.7	**C5259**	-4.1	**A8598**	-3.7	**A8981**	-3.3	**N3288**	-2.9	**I2892**	-2.0
**R-115**	-7.2	**C5270**	-4.7	**C8145**	-4.1	**A9950**	-3.7	**B7273**	-3.3	**PZ0140**	-2.9	**SML0040**	-2.0
**265128**	-7.0	**C7861**	-4.7	**P-178**	-4.1	**B6436**	-3.7	**C1493**	-3.3	**T-103**	-2.9	**T9033**	-2.0
**A0606 (*)**	-6.9, 15.2	**C8395**	-4.7	**P-215**	-4.1	**D-103**	-3.7	**D-031**	-3.3	**T-144**	-2.9	**L8668**	-2.0
**H5257**	-6.9	**C8903**	-4.7	**P7340**	-4.1	**D-131**	-3.7	**D-122**	-3.3	**T2879**	-2.9	**N2001**	-2.0
**A6566**	-6.7	**S7701**	-4.6	**P8688**	-4.1	**D-193**	-3.7	**D-134**	-3.3	**T6050**	-2.9	**N5504**	-2.0
**A7162**	-6.5	**A5922**	-4.6	**P9297**	-4.1	**D9128**	-3.7	**D14204**	-3.3	**G8761**	-2.9	**N5636**	-2.0
**246379**	-6.5	**A-201**	-4.6	**P9797**	-4.1	**E3256**	-3.7	**D7910**	-3.3	**N7510**	-2.9	**P0453**	-2.0
**246557**	-6.5	**A-230**	-4.6	**PZ0210**	-4.1	**F6886**	-3.7	**E0516**	-3.3	**P2607**	-2.9	**P0884**	-2.0
**A3085**	-6.5	**A-254**	-4.6	**Q0125**	-4.1	**K4144**	-3.7	**E2387**	-3.3	**P4543**	-2.9	**P4394**	-2.0

Cat No	ΔT_m	Cat No	ΔT_m	Cat No	ΔT_m	Cat No	ΔT_m	Cat No	ΔT_m	Cat No	ΔT_m	Cat No	ΔT_m
**A5626**	-6.4	**A-255**	-4.6	**S0693**	-4.1	**L6668**	-3.7	**E3132**	-3.3	**C4024**	-2.9	**SML0234**	-2.0
**I-119**	-6.4	**A-263**	-4.6	**S2816**	-4.1	**PZ0014**	-3.7	**E3876**	-3.3	**C6506**	-2.9	**E-006**	-1.9
**M2547**	-6.4	**A8723**	-4.6	**SML0524**	-4.1	**S-009**	-3.7	**F8927**	-3.3	**C9758**	-2.9	**F6300**	-1.9
**A0430**	-6.4	**A9501**	-4.6	**SML0704**	-4.1	**S0568**	-3.7	**N2288**	-3.3	**P7791**	-2.9	**PZ0196**	-1.9
**O7639**	-6.4	**C1240**	-4.6	**B-112**	-4.0	**S-106**	-3.7	**X1251**	-3.3	**PZ0113**	-2.9	**L9793**	-1.9
**PZ0162**	-6.4	**F-131**	-4.6	**B2390**	-4.0	**SML0134**	-3.7	**C-199**	-3.3	**Q-110**	-2.9	**N1016**	-1.9
**H2380**	-6.3	**G2128**	-4.6	**B-5311**	-4.0	**T-123**	-3.7	**C9911**	-3.3	**R-108**	-2.9	**N2034**	-1.9
**861804**	-6.3	**H-127**	-4.6	**B7005**	-4.0	**T4376**	-3.7	**D0670**	-3.3	**SML0752**	-2.9	**N3136**	-1.9
**PZ0001**	-6.3	**PZ0022**	-4.6	**B7777**	-4.0	**T6376**	-3.7	**D1916**	-3.3	**T7080**	-2.9	**P1061**	-1.9
**A5376**	-6.2	**S-168**	-4.6	**B8385**	-4.0	**T7313**	-3.7	**D3630**	-3.3	**C1610**	-2.8	**T0826**	-1.9
**PZ0020**	-6.2	**T1698 (#)**	-4.6, -17.2	**C0330**	-4.0	**T8516**	-3.7	**L8401**	-3.3	**PZ0171**	-2.8	**L3169**	-1.9
**A0382**	-6.2	**T2896**	-4.6	**C1671**	-4.0	**U-101**	-3.7	**M6545**	-3.3	**C0256**	-2.8	**SML0720**	-1.9
**D5446**	-6.2	**T7540**	-4.6	**S7067**	-4.0	**W-104**	-3.7	**M7445**	-3.3	**D9305**	-2.8	**B-121**	-1.8
**T3955**	-6.1	**D3775**	-4.6	**SML0564**	-4.0	**Z0878**	-3.7	**PZ0104**	-3.3	**P0115**	-2.8	**M-226**	-1.8
**A-265**	-6.1	**D4526**	-4.6	**A-022**	-4.0	**Z2001**	-3.7	**T0202**	-3.3	**SML0218**	-2.8	**F0778**	-1.7
**A4393**	-6.0	**A0384**	-4.6	**A-145**	-4.0	**D1413**	-3.7	**M-110**	-3.3	**SML0229**	-2.8	**F6145**	-1.7
**B7283**	-6.0	**C3912**	-4.6	**A8456**	-4.0	**D1507**	-3.7	**M-129**	-3.3	**T7883**	-2.8	**G6649**	-1.7
**M152**	-6.0	**C4397**	-4.6	**B0753**	-4.0	**G5793**	-3.7	**N-151**	-3.3	**T9778**	-2.8	**B6938**	-1.6
**P7912**	-6.0	**C5020**	-4.6	**B9311**	-4.0	**H1877**	-3.7	**N4148**	-3.3	**U7500**	-2.8	**E8875**	-1.6
**A4910**	-5.9	**C6019**	-4.6	**C-125**	-4.0	**I4883**	-3.7	**O2378**	-3.3	**V8261**	-2.8	**SML0536**	-1.6
**T9034**	-5.9	**C7041**	-4.6	**D5689**	-4.0	**I9890**	-3.7	**O3011**	-3.3	**W1628**	-2.8	**T8543**	-1.6
**A9561**	-5.9	**O111**	-4.6	**D5891**	-4.0	**L-109**	-3.7	**P2016**	-3.3	**Y-102**	-2.8	**A0233**	-1.6
**T3146**	-5.9	**SML0221**	-4.6	**D8296**	-4.0	**L-121**	-3.7	**PZ0213**	-3.3	**B9685**	-2.8	**A6605**	-1.6
**P8765**	-5.9	**B3023**	-4.5	**G-117**	-4.0	**L9908**	-3.7	**B4311**	-3.3	**C-271**	-2.8	**A5879**	-1.5
**D6518**	-5.8	**B3501**	-4.5	**G-154**	-4.0	**M4659**	-3.7	**P-120**	-3.3	**D3900**	-2.8	**A6134**	-1.5
**291552**	-5.8	**B5681**	-4.5	**H0879**	-4.0	**M6690**	-3.7	**P-162**	-3.3	**D5294**	-2.8	**A6733**	-1.5
**A7148**	-5.7	**C1625**	-4.5	**H-135**	-4.0	**PZ0100**	-3.7	**P8293**	-3.3	**L-135**	-2.8	**N0630**	-1.5
**A-023**	-5.7	**D-047**	-4.5	**H5752**	-4.0	**SML0264**	-3.7	**P8828**	-3.3	**S9311**	-2.8	**P0547**	-1.5

Cat No	ΔT_m	Cat No	ΔT_m	Cat No	ΔT_m	Cat No	ΔT_m	Cat No	ΔT_m	Cat No	ΔT_m	Cat No	ΔT_m
**B8312**	-5.7	**D3768**	-4.5	**H9882**	-4.0	**SML0667**	-3.7	**P8887**	-3.3	**SML0247**	-2.8	**M7033**	-1.4
**D3689**	-5.7	**F6889**	-4.5	**I7379**	-4.0	**U6881**	-3.7	**P8891**	-3.3	**C4915**	-2.8	**R0529**	-1.4
**D5564**	-5.7	**G3126**	-4.5	**I9531**	-4.0	**C3130**	-3.7	**P9178**	-3.3	**E5406**	-2.8	**N2255**	-1.4
**A3134**	-5.7	**H-168**	-4.5	**L2536**	-4.0	**C3412**	-3.7	**R6250**	-3.3	**N4159**	-2.8	**P1675**	-1.4
**E-100**	-5.6	**I7388**	-4.5	**L3791**	-4.0	**C8088**	-3.7	**S0441**	-3.3	**O3636**	-2.8	**R1402**	-1.4
**G-119**	-5.6	**M1275**	-4.5	**L4762**	-4.0	**C8138**	-3.7	**S8251**	-3.3	**P2116**	-2.8	**R8875**	-1.4
**SML0527**	-5.6	**T4512**	-4.5	**L5025**	-4.0	**C9510**	-3.7	**B6311**	-3.2	**R7772**	-2.8	**A5006**	-1.3
**T6951**	-5.6	**A0760**	-4.5	**L9756**	-4.0	**C9754**	-3.7	**R1283**	-3.2	**A0779**	-2.8	**I7378**	-1.3
**C5793**	-5.6	**A1260**	-4.5	**M1809**	-4.0	**E2535**	-3.7	**A-236**	-3.2	**R-116**	-2.8	**M0814**	-1.3
**C8011**	-5.6	**G5918**	-4.5	**M5171**	-4.0	**M-184**	-3.7	**A4233**	-3.2	**R-134**	-2.8	**H9415**	-1.2
**A6351**	-5.5	**N8652**	-4.5	**M5435**	-4.0	**O0766**	-3.7	**A5181**	-3.2	**R3255**	-2.8	**E7881**	-1.1
**SML0505**	-5.5	**P5514**	-4.5	**M5560**	-4.0	**P0878**	-3.7	**D-030**	-3.2	**S1438**	-2.8	**D8941**	-1.0
**I1149**	-5.5	**P7136**	-4.5	**M5685**	-4.0	**S3442**	-3.7	**D-052**	-3.2	**S1875**	-2.8	**L-106**	-1.0
**SML0113**	-5.5	**A3940**	-4.4	**M7277**	-4.0	**A0500**	-3.7	**D-054**	-3.2	**S8502**	-2.8	**A9480 (#)**	-0.8, -11.3
**T7165**	-5.5	**A5330**	-4.4	**PZ0003**	-4.0	**I0658**	-3.7	**E0137**	-3.2	**PZ0185**	-2.7	**I2285**	-0.8
**A1895**	-5.5	**A8001**	-4.4	**PZ0015**	-4.0	**P-102**	-3.7	**N1415**	-3.2	**A7111**	-2.7	**H8653**	-0.6
**A1910**	-5.5	**B-169**	-4.4	**PZ0139**	-4.0	**P8782**	-3.7	**N3911**	-3.2	**B7688**	-2.7	**N3510**	-0.6
**A5282**	-5.4	**B8279**	-4.4	**S-174**	-4.0	**PZ0110**	-3.7	**S-153**	-3.2	**D6140**	-2.7	**P1793**	-0.6
**A5909**	-5.4	**S9318**	-4.4	**SML0517**	-4.0	**Q1250**	-3.7	**S5317**	-3.2	**D8555 (*)**	-2.7, 27.7	**S4250**	-0.6
**D9628**	-5.4	**E4642**	-4.4	**SML0634**	-4.0	**Q3504**	-3.7	**T0254**	-3.2	**E4378**	-2.7	**G6423**	-0.1
**H7779**	-5.4	**F9552**	-4.4	**SML0644**	-4.0	**S7389**	-3.7	**T-200**	-3.2	**PZ0115**	-2.7	**I8898**	0.0
**I-120**	-5.4	**G2536 (*)**	-4.4, 11.5	**SML0841**	-4.0	**S8139**	-3.7	**T7947**	-3.2	**SML0075**	-2.7	**SML0245**	0.0
**P-152**	-5.4	**G9797**	-4.4	**T0410**	-4.0	**B2377**	-3.6	**W2270**	-3.2	**SML0601**	-2.7	**T2705**	0.0
**SML0255**	-5.4	**A-202**	-4.4	**T1694**	-4.0	**B6506**	-3.6	**C5493**	-3.2	**SML0892**	-2.7	**SML0209**	0.0
**SML0658**	-5.4	**A7762**	-4.4	**T6394**	-4.0	**B7651**	-3.6	**H7250**	-3.2	**T2067**	-2.7	**D5794**	6.2

Cat No	ΔT_m	Cat No	ΔT_m	Cat No	ΔT_m	Cat No	ΔT_m	Cat No	ΔT_m	Cat No	ΔT_m	Cat No	ΔT_m
**M8046**	-5.4	**A7845**	-4.4	**T7040**	-4.0	**C2755**	-3.6	**I1637**	-3.2	**T2408**	-2.7	**C3270 (*)**	-2.9, 12.1
**N-144**	-5.4	**A9345**	-4.4	**V8879**	-4.0	**A6011**	-3.6	**I6504**	-3.2	**T3757**	-2.7	**C3662 (*)**	-3.2, 14.8
**144509**	-5.4	**B5437**	-4.4	**X6000**	-4.0	**PZ0002**	-3.6	**I8021**	-3.2	**U-120**	-2.7	**SML0476 (*)**	-2.7, 16.6
**A1755**	-5.4	**C-239**	-4.4	**Z4902**	-4.0	**A-013**	-3.6	**L1011**	-3.2	**U4125**	-2.7	**C7522 (*)**	-4.0, 22.3
**S5890**	-5.4	**D-027**	-4.4	**C4479**	-4.0	**A-143**	-3.6	**L-119**	-3.2	**V-100**	-2.7	**SML0282 (*)**	-3.2, 23.3
**A6671**	-5.3	**D1414**	-4.4	**C4522**	-4.0	**A-164**	-3.6	**M1514**	-3.2	**D0540**	-2.7	**SML0551 (*)**	-3.2, 31.9
**A9512**	-5.3	**D2521**	-4.4	**C4895**	-4.0	**D8399**	-3.6	**M5154**	-3.2	**H1753**	-2.7		
**F6800**	-5.3	**D4505**	-4.4	**C4911**	-4.0	**D9035**	-3.6	**M5250**	-3.2	**S4572**	-2.7		
**V8138**	-5.3	**D5782**	-4.4	**C5982**	-4.0	**F1553**	-3.6	**B2185**	-3.2	**SML0586**	-2.7		
**C0494**	-5.3	**D5886**	-4.4	**C6643**	-4.0	**F6020**	-3.6	**L4545**	-3.2	**M9651**	-2.7		
**C-145**	-5.3	**F4429**	-4.4	**C7255**	-4.0	**F9397**	-3.6	**M9020**	-3.2	**N1530**	-2.7		
**F8682**	-5.3	**F7932**	-4.4	**C7971**	-4.0	**H-140**	-3.6	**N-142**	-3.2	**N-170**	-2.7		
**H7258**	-5.3	**H1384**	-4.4	**P-103**	-4.0	**M6383**	-3.6	**N7505**	-3.2	**N4163**	-2.7		
**H8502**	-5.3	**H4759**	-4.4	**P63204**	-4.0	**P0111**	-3.6	**N9007**	-3.2	**N7758**	-2.7		
**H8876**	-5.3	**I7627**	-4.4	**P8477**	-4.0	**PZ0136**	-3.6	**P4532**	-3.2	**P0778**	-2.7		
**H9003**	-5.3	**J4137**	-4.4	**P9879**	-4.0	**S-154**	-3.6	**P6126**	-3.2	**P4668**	-2.7		
**M1404**	-5.3	**M1387**	-4.4	**Q0875**	-4.0	**S-180**	-3.6	**P6628**	-3.2	**T5575**	-2.7		
**A1784**	-5.3	**M7320**	-4.4	**SML0678**	-4.0	**SML0653**	-3.6	**SML0227**	-3.2	**A0937**	-2.7		
**A7127**	-5.2	**P9248**	-4.4	**A4147**	-3.9	**T0891**	-3.6	**SML0552**	-3.2	**R-121**	-2.7		
**PZ0004**	-5.2	**S3572**	-4.4	**B-161**	-3.9	**T1516**	-3.6	**SML0630**	-3.2	**R7150**	-2.7		
**A9899**	-5.2	**U-111**	-4.4	**B-168**	-3.9	**T1633 (*)**	-3.6, 6.0	**A8231**	-3.2	**R8900**	-2.7		

Cat No	ΔT_m	Cat No	ΔT_m	Cat No	ΔT_m	Cat No	ΔT_m	Cat No	ΔT_m	Cat No	ΔT_m	Cat No	ΔT_m
**B1381**	-5.2	**N1392**	-4.4	**B2134**	-3.9	**T4182**	-3.6	**C1494**	-3.2	**S7882**	-2.7		
**E7906**	-5.2	**P0667**	-4.4	**B7880**	-3.9	**W-108**	-3.6	**P-101**	-3.2	**S8442**	-2.7		
**F4765**	-5.2	**P6503**	-4.4	**C0400**	-3.9	**Z-101**	-3.6	**R2625**	-3.2	**T6580**	-2.7		
**P0122**	-5.2	**S6319**	-4.4	**C0424**	-3.9	**D1306**	-3.6	**R3277**	-3.2	**D9175**	-2.6		
**S-143**	-5.2	**A0152**	-4.4	**PZ0158**	-3.9	**C4042**	-3.6	**S3378**	-3.2	**D9446**	-2.6		
**C-104**	-5.2	**A0487**	-4.4	**SML0492**	-3.9	**C5923**	-3.6	**B-016**	-3.1	**I9532**	-2.6		
**H0131**	-5.2	**A0966**	-4.4	**E-101**	-3.9	**C6022**	-3.6	**B-138**	-3.1	**K1136**	-2.6		
**D9891**	-5.1	**C7897**	-4.4	**A-242**	-3.9	**C7632**	-3.6	**B5683**	-3.1	**K3375**	-2.6		
**E8375**	-5.1	**R0758**	-4.4	**C1618**	-3.9	**C8031**	-3.6	**B9308**	-3.1	**M0253**	-2.6		
**G-133**	-5.1	**A6883**	-4.3	**C-191**	-3.9	**D6321**	-3.6	**SML0520**	-3.1	**M7945**	-2.6		
**H9002**	-5.1	**B-135**	-4.3	**D1064**	-3.9	**D8696**	-3.6	**A3773**	-3.1	**O7389**	-2.6		
**I-122**	-5.1	**B5016**	-4.3	**D5766**	-3.9	**I5409**	-3.6	**A9605**	-3.1	**R8404**	-2.6		
**M2727**	-5.1	**PZ0142**	-4.3	**D7644**	-3.9	**M-149**	-3.6	**D-002**	-3.1	**S9186**	-2.6		
**P0099**	-5.1	**SML0189**	-4.3	**D9571**	-3.9	**O3752**	-3.6	**D126608**	-3.1	**SML0275**	-2.6		
**C-130**	-5.1	**A-178**	-4.3	**E1779**	-3.9	**P7412**	-3.6	**D-129**	-3.1	**T9025**	-2.6		
**A0257**	-5.1	**A7250**	-4.3	**E9531**	-3.9	**P7561**	-3.6	**D7505**	-3.1	**X4753**	-2.6		
**B8262**	-5.0	**A7824**	-4.3	**G-017**	-3.9	**PZ0116**	-3.6	**D8008**	-3.1	**C-192**	-2.6		
**C0625**	-5.0	**D7443**	-4.3	**G3796**	-3.9	**R-103**	-3.6, -19,4	**D9190**	-3.1	**A9861**	-2.6		
**C1172**	-5.0	**E5156**	-4.3	**G7795**	-3.9	**R6152**	-3.6	**D9766**	-3.1	**I7160**	-2.6		
**F3680**	-5.0	**F-124**	-4.3	**I-135**	-3.9	**T6950**	-3.6	**H0126**	-3.1	**M3071**	-2.6		
**A2729**	-5.0	**G8134**	-4.3	**I-146**	-3.9	**A4669**	-3.5	**H8627**	-3.1	**N7261**	-2.6		
**A8835**	-5.0	**O5889**	-4.3	**I3639**	-3.9	**C0737**	-3.5	**I6659**	-3.1	**N7778**	-2.6		

Cat No	ΔT_m	Cat No	ΔT_m	Cat No	ΔT_m	Cat No	ΔT_m	Cat No	ΔT_m	Cat No	ΔT_m	Cat No	ΔT_m
**C9901**	-5.0	**C3118**	-4.3	**L9787**	-3.9	**C0987**	-3.5	**I8250**	-3.1	**O8757**	-2.6		
**D-003**	-5.0	**D0676**	-4.3	**M2901**	-3.9	**A6664**	-3.5	**L0664**	-3.1	**O9126**	-2.6		
**D2531**	-5.0	**D5439**	-4.3	**M3127**	-3.9	**A9013**	-3.5	**L2411**	-3.1	**P4509**	-15,6		
**E3263**	-5.0	**H1252**	-4.3	**M3184**	-3.9	**C-101**	-3.5	**L5647**	-3.1	**P4670**	-2.6		
**F4646**	-5.0	**H2775**	-4.3	**M4145**	-3.9	**C-102**	-3.5	**M1559**	-3.1	**P6402**	-2.6		
**H9772**	-5.0	**H6036**	-4.3	**M5441**	-3.9	**C-117**	-3.5	**M4910**	-3.1	**T7205**	-2.6		
**I2760**	-5.0	**I0157**	-4.3	**M6760**	-3.9	**C-147**	-3.5	**M5391**	-3.1	**T8703**	-2.6		
**L2167**	-5.0	**I-114**	-4.3	**M7319**	-3.9	**C-203**	-3.5	**M6500**	-3.1	**X3629**	-2.6		
**P9623**	-5.0	**I-117**	-4.3	**M7684**	-3.9	**C-237**	-3.5	**S-008**	-3.1	**C-197**	-2.5		
**SML0015**	-5.0	**L6545**	-4.3	**N0287**	-3.9	**D0411**	-3.5	**S4443**	-3.1	**D3634**	-2.5		
**T1132**	-5.0	**M2525**	-4.3	**P0618**	-3.9	**D1262**	-3.5	**SML0226**	-3.1	**D4007**	-2.5		
**A1824**	-5.0	**M2537**	-4.3	**PZ0107**	-3.9	**D-138**	-3.5	**SML0613**	-3.1	**D6899**	-2.5		
**C7291**	-5.0	**PZ0013**	-4.3	**PZ0114**	-3.9	**D2926**	-3.5	**U6758**	-3.1	**E2031**	-2.5		
**P6909**	-5.0	**T1505**	-4.3	**PZ0137**	-3.9	**D7938**	-3.5	**Y3125**	-3.1	**E3380**	-2.5		
**R0875**	-5.0	**T4143**	-4.3	**PZ0141**	-3.9	**E0381**	-3.5	**C-108**	-3.1	**E4375**	-2.5		
**A0232**	-4.9	**T4425**	-4.3	**S4528**	-3.9	**E1896**	-3.5	**D1260**	-3.1	**S8688**	-2.5		
**F2802**	-4.9	**C7005**	-4.3	**S5192**	-3.9	**E2375**	-3.5	**D0943**	-3.1	**SML0610**	-2.5		
**A9834**	-4.9	**C8645**	-4.3	**T1443**	-3.9	**F6513**	-3.5	**N3398**	-3.1	**SML0824**	-2.5		
**C6492**	-4.9	**M5793**	-4.3	**T6031**	-3.9	**G0668**	-3.5	**N3529**	-3.1	**SML0864**	-2.5		
**G-002**	-4.9	**N8534**	-4.3	**U-105**	-3.9	**H1512**	-3.5	**N7634**	-3.1	**B5435**	-2.5		
**G0419**	-4.9	**P1918**	-4.3	**U5882**	-3.9	**I-138**	-3.5	**O-100**	-3.1	**M-001**	-2.5		
**H0627**	-4.9	**P5396**	-4.3	**V4629**	-3.9	**I5627**	-3.5	**O2881**	-3.1	**N-153**	-2.5		

Cat No	ΔT$_m$	Cat No	ΔT$_m$	Cat No	ΔT$_m$	Cat No	ΔT$_m$	Cat No	ΔT$_m$	Cat No	ΔT$_m$	Cat No	ΔT$_m$
**H8125**	-4.9	**PZ0111**	-4.3	**V6383**	-3.9	**L-131**	-3.5	**O9637**	-3.1	**N-183**	-2.5		
**I0375**	-4.9	**P-203**	-4.3	**N8403**	-3.9	**L4376**	-3.5	**P-105**	-3.1	**N4382**	-2.5		
**I2279**	-4.9	**P8813**	-4.3	**N8659**	-3.9	**L8533**	-3.5	**P1784**	-3.1	**P2278**	-2.5		
**I4409 (#)**	-4.9, -19,7	**R5648**	-4.3	**P5295**	-3.9	**M1777**	-3.5	**P1801**	-3.1	**PZ0102**	-2.5		
**L4900**	-4.9	**B2417**	-4.2	**P5654**	-3.9	**M5644**	-3.5	**P4651**	-3.1	**R2530**	-2.5		
**M6191**	-4.9	**G6548**	-4.2	**A1237**	-3.9	**M6524**	-3.5	**P6499**	-3.1	**S2201**	-2.5		
**SML02 16**	-4.9	**A8404**	-4.2	**A1980**	-3.9	**M6628**	-3.5	**P7295**	-3.1	**S3065**	-2.5		
**SML02 69**	-4.9	**C9369**	-4.2	**B5063**	-3.9	**O1141**	-3.5	**P8852**	-3.1	**SML0683**	-2.5		
**SML06 60**	-4.9	**D-108**	-4.2	**P6656**	-3.9	**PZ0016**	-3.5	**PZ0101**	-3.1	**A4562**	-2.4		
**N5023**	-4.9	**D5676**	-4.2	**P6777**	-3.9	**S-006**	-3.5	**PZ0117**	-3.1	**B-134**	-2.4		
**P3510**	-4.9	**D6035**	-4.2	**P8013**	-3.9	**SML0223**	-3.5	**R0500**	-3.1	**C0862**	-2.4		
**194336**	-4.9	**F-114**	-4.2	**P9233**	-3.9	**T0625**	-3.5	**S5321**	-3.1	**F4303**	-2.4		
**211672**	-4.9	**F9677**	-4.2	**P9547**	-3.9	**T-104**	-3.5	**SML0711**	-3.1	**F8257**	-2.4		
**A3145**	-4.9	**T2057**	-4.2	**PZ0112**	-3.9	**T6943**	-3.5	**B-102**	-3.0	**N2538**	-2.4		
**C3930**	-4.9	**V1377**	-4.2	**R9525**	-3.9	**V7264**	-3.5	**C2538**	-3.0	**PZ0012**	-2.4		
**C6895**	-4.9	**G-007**	-4.2	**S2812**	-3.9	**C8773**	-3.5	**D3943**	-3.0	**SML0639**	-2.4		
**G5794**	-4.9	**H8645**	-4.2	**S7936**	-3.9	**E9658**	-3.5	**A1362**	-3.0	**T5648**	-2.4		
**P6902**	-4.9	**H9876**	-4.2	**SML0550**	-3.9	**H8664**	-3.5	**B6813**	-3.0	**D2763**	-2.4		
**Q-109**	-4.9	**I-106**	-4.2	**Z2777**	-3.9	**P-118**	-3.5	**C0996**	-3.0	**H4001**	-2.4		
**S7947**	-4.9	**I7016**	-4.2	**C2235**	-3.8	**P7780**	-3.5	**C1119**	-3.0	**T-122**	-2.4		
**A4638**	-4.8	**M0763**	-4.2	**A-155**	-3.8	**P8511**	-3.5	**C3618**	-3.0	**Y0503**	-2.4		

Cat No	ΔT_m	Cat No	ΔT_m	Cat No	ΔT_m	Cat No	ΔT_m	Cat No	ΔT_m	Cat No	ΔT_m	Cat No	ΔT_m
B4555	-4.8	M1818	-4.2	A6476	-3.8	PZ0108	-3.5	D-104	-3.0	M-104	-2.4		
C1159	-4.8	M2398	-4.2	A8676	-3.8	Q3251	-3.5	F0881	-3.0	N5501	-2.4		
A-129	-4.8	PZ0211	-4.2	A9251	-3.8	R-104	-3.5	F-100	-3.0	O0250	-2.4		
A8003	-4.8	SML0091	-4.2	A9809	-3.8	R7385	-3.5	F1016	-3.0	O1008	-2.4		
A9256	-4.8	SML0130	-4.2	C8863	-3.8	R9644	-3.5	G6416	-3.0	P4015	-2.4		
B2009	-4.8	SML0179	-4.2	D-1920-6	-3.8	S2876	-3.5	I-160	-3.0	SML0612	-2.4		
E3149	-4.8	C3635	-4.2	D8040	-3.8	S5013	-3.5	I18008	-3.0	R-140	-2.4		
G0639	-4.8	C4238	-4.2	H3288	-3.8	S8197	-3.5	K0250	-3.0	T2952	-2.4		
R0158	-4.8	C5134	-4.2	PZ0178	-3.8	A3539	-3.4	K1751	-3.0	Y4877	-2.3		
S3316	-4.8	C6645	-4.2	SML0594	-3.8	B2292	-3.4	L0258	-3.0	A8852	-2.3		
SML0776	-4.8	M-137	-4.2	T9652	-3.8	C1754	-3.4	L4408	-3.0	D6940	-2.3		
T-101	-4.8	N-115	-4.2	C-141	-3.8	S3322	-3.4	M2776	-3.0	F6627	-2.3		
A7606	-4.8	O3125	-4.2	C-223	-3.8	A8054	-3.4	M3262	-3.0	G4796	-2.3		
H-108	-4.8	P6500	-4.2	D1542	-3.8	C-144	-3.4	M3281	-3.0	PZ0008	-2.3		
H1377	-4.8	A2129	-4.2	I2764	-3.8	C-231	-3.4	M3668	-3.0	B175	-2.3		
N1786	-4.8	P7505	-4.2	J4829	-3.8	D5290	-3.4	M3953	-3.0	H8250	-2.3		
T2528	-4.8	P8139	-4.2	L9664	-3.8	D5385	-3.4	M4796	-3.0	I3766	-2.3		
C6048	-4.8	B2640	-4.1	M7065	-3.8	E1383	-3.4	PZ0011	-3.0	M-105	-2.3		
C6628	-4.8	B3650	-4.1	SML0679	-3.8	K3888	-3.4	PZ0121	-3.0	M-107	-2.3		
C7912	-4.8	B5275	-4.1	SML0777	-3.8	L2037	-3.4	S3567	-3.0	M9292	-2.3		
I0160	-4.8	B5399	-4.1	T1512	-3.8	L2906	-3.4	S5567	-3.0	M9656	-2.3		
M9511	-4.8	C0750	-4.1	T4264	-3.8	L5783	-3.4	S8822	-3.0	N-158	-2.3		

Cat No	ΔT$_m$	Cat No	ΔT$_m$	Cat No	ΔT$_m$	Cat No	ΔT$_m$	Cat No	ΔT$_m$	Cat No	ΔT$_m$	Cat No	ΔT$_m$
**PZ0135**	-4.8	**C1251**	-4.1	**T4500**	-3.8	**M4531**	-3.4	**SML0817**	-3.0	**O0877**	-2.3		
**218359**	-4.8	**C1290**	-4.1	**T5576**	-3.8	**M5379**	-3.4	**T7665**	-3.0	**PZ0124**	-2.3		
**SML0511 (#)**	-4.8, -21,8	**A4508**	-4.1	**T5625**	-3.8	**M6517**	-3.4	**U-103**	-3.0	**SML0246**	-2.3		
**A3711**	-4.7	**A-138**	-4.1	**T6764**	-3.8	**SML0233**	-3.4	**U-104**	-3.0	**E9750**	-2.2		
**A7009**	-4.7	**A-162**	-4.1	**C4542**	-3.8	**T0318**	-3.4	**U-106**	-3.0	**F8791**	-2.2		
**A7410**	-4.7	**D7909**	-4.1	**C7230**	-3.8	**T6164**	-3.4	**Y-101**	-3.0	**SML0252**	-2.2		
**A9657**	-4.7	**D9815**	-4.1	**L5420**	-3.8	**T7254**	-3.4	**C6305**	-3.0	**T9262**	-2.2		
**A9699**	-4.7	**E4660**	-4.1	**M2199**	-3.8	**U-115**	-3.4	**C8221**	-3.0	**K3519**	-2.2		
**B0385**	-4.7	**F9427**	-4.1	**P5052**	-3.8	**Z1252**	-3.4	**D-033**	-3.0	**M-116**	-2.2		
**B1266**	-4.7	**G-110**	-4.1	**120693**	-3.8	**M-108**	-3.4	**F0430**	-3.0	**M4699**	-2.2		
**C6617**	-4.7	**H-128**	-4.1	**A2169**	-3.8	**M8131**	-3.4	**K1015**	-3.0	**M9125**	-2.2		
**F-132**	-4.7	**H9523**	-4.1	**G9048**	-3.8	**N-149**	-3.4	**P9391**	-3.0	**N-156**	-2.2		
**G6793**	-4.7	**I5879**	-4.1	**P-126**	-3.8	**N8784**	-3.4	**PZ0192 (*)**	-3.0, 10.3	**N4779**	-2.2		
**N2540**	-4.7	**L8539**	-4.1	**P-154**	-3.8	**O0886**	-3.4	**S0758**	-3.0	**N5751**	-2.2		
**PZ0005**	-4.7	**P9872**	-4.1	**P8386**	-3.8	**P5679**	-3.4	**S2501**	-3.0	**N7127**	-2.2		
**PZ0007**	-4.7	**PZ0106**	-4.1	**PZ0103**	-3.8	**C3025**	-3.4	**SML0518**	-3.0	**O2751**	-2.2		
**S2671**	-4.7	**SML0191**	-4.1	**Q-102**	-3.8	**I5784**	-3.4	**SML0557**	-3.0	**P0130**	-2.2		
**S8567**	-4.7	**SML0210**	-4.1	**Q2128**	-3.8	**R-101**	-3.4	**Z4626**	-3.0	**P4405**	-2.2		

Table S4: Compounds tested in previous clinical trials of Phase II or III in ALS patients with inconclusive results or failure to demonstrate significantly importance or implications with safety. Including those by efficacy in studies from SOD1 murine models. (Those carried out before 1993 are not related to SOD1).

Compound	Reference and year	(speculated) mechanism /comments
**Ceftriaxone**	[3,4,5]	Neuroprotection related to mediating glutamate toxicity by increasing expression of EAAT2 an its biochemical and functional activity in SOD1(G93A) animal models [6].<br><br>Guo et al. noticed a reduction in SOD1 aggregates in EAAT2/G93A double tg mice (not through protein-compound interaction) [5]. Toxicity issues during CT [4].
**Talampanel (8-methyl-7H-1,3-dioxolo(2,3)benzodiazepine)**	[7]	Probably implicated with excitotoxicity mechanisms through ionotropic glutamate receptors (that are activated by glutamate).
**Tamoxifen**	[8,9]	Attenuates TDP-43 aggregation and enhances autophagy.
**Minocycline**	[10,11]	Anti-apoptotic and anti-inflammatory effects in vitro. Proceeded to III with contradictory results!<br><br>Inhibits activity of caspase-1, caspase-3, microglial activation, inducible nitric oxide synthetase up-regulation and inhibits mitochondrial cytochrome c release, all related to ALS [12,13].<br><br>It delayed disease onset and extended survival in SOD1(G93A) tg mice [12,13] and G37R [14].
**TCH346**	[15]	Antiapoptotic effect by binding to glyceraldehyde 3-phosphate dehydrogenase (GAPDH) and blocking its apoptotic pathway.
**Coenzyme Q10**	[16]	Antioxidant and mitochondrial co-factor Promising data from tg mice [17].
**Vitamin E**	[18–20]	Antioxidant. In SOD mice model [21] delays onset of clinical disease and slows progression.
**Celecoxib**	[22]	COX-2 inhibitor agent for arthritis that blocks prostaglandin synthesis. Related with anti-inflammatory action. In SOD1(G93A) tg mouse model delayed the onset of weakness and prolonged survival [23].
**Creatine**	[24,25]	Related to energy metabolism and may attenuate mitochondrial dysfunction. Prolonged survival in mice [26].
**Copaxone**	[27]	Immunomodulatory through interaction of T-cells with microglial cells (prolongs expectancy in SOD mice [28].
**Insulin-like growth factor (IGF-1, ormyotrophin)**	[29]	In SOD1 mice [30] protects mitochondria from apoptosis and upregulates mitophagy.
**brain derived neurotrophic factor, ciliary neurotrophic factor, glial cell line derived neurotrophic factor, xaliproden**	[15,31,32, 33]	Neurotrophic effect.
**ONO-2506 (homologue of valproic acid)**	[34, 35]	Antioxidative and antiapoptotic properties.<br><br>In SOD1 mice prolongs survival [36].

Compound	Reference and year	(speculated) mechanism /comments
**valproate/lithium**	[37,38]	In SOD1(G93A) mice delayed the onset of symptoms, prolonges lifespan and decreased neurological deficit scores [39]. The trial stopped.
**Lithium**	[40]	The trial stopped.
**CK-2017357 (Tirasemtiv)**	[41,42]	Fast skeletal muscle troponin activator.<br><br>Proceeded to III.
**KNS-760704 (dexpramipexole)**	[43, 44,45]	Neuroprotection in SOD1G93A mouse model, maintaining mitochondrial function. Proceeded to trial III.
**Methylcobalamin (vit B12)**	[46,47]	Neuroprotective effect: acts as a coenzyme by donating methyl in homocysteine remethylation, reducing homocysteine levels related to neurodegeneration [48]. The second trial used ultra-high dose and showed more promising results.
**Topiramate**	[49]	Antioxidant.
**Phenylbutyrate sodium**	[50]	Transcription regulation, improvement of PT pathways. In tg mice reduced SOD1-positive aggregates and dosage-dependently prolonged survival [51].
**Statins**	[52]	Neuroprotective-immunomodulatory effect.
**Indinavir**	[53]	Other-toxicity.
**Verapamil**	[54]	Calcium regulation. In SOD1(G93A) mice delaying onset of ALS and prolonging lifespan. Improved autophagic flux, reduced SOD1 protein aggregation, ameliorating ER stress and inhibiting glia activation [55].
**Nimodipine**	[56]	Calcium regulation.
**Branched-Chain Aminoacids**	[57]	Energy metabolism.
**thyrotropin releasing hormone (TRH)**	[58]	Trophic factor.
**Octacosanol**	[59]	Anti-iflammatory
**Tilorone**	[60]	Other
**Isoprinosine**	[61]	Other
**Cyclosporin**	[62]	Keep et al. using a SOD1(G93A) tg mice validated neuroprotective action when it crosses the BBB. Its administration prolonged survival of late stage ALS tg animals, relating its mechanism of action to mitochondrial function [63].
**GABApentin**	[64,65]	Possible presynaptic inhibitor of glutamate biosynthesis.
**Pentoxifylline**	[66]	Other
**Ravulizumab/Zilucoplan**	(NCT04248465)/(NCT04297683, NCT04436497), respectively	Targets complement cascade (part of immune system).
**Levosimendan**	(NCT03948178)	Binds to troponin C and sensitizes cardiac and skeletal muscle to calcium.

Compound	Reference and year	(speculated) mechanism /comments
**Ropinirol**	[67]	Used to treat PD.
**Dexpramipexole**	NCT01281189	Used to treat PD.
**Ezogabine (Retigabine)**	NCT02450552	Other
**Thalidomide**	[68]	Immunomodulatory agent: inhibits the expression of TNF-alfa and other cytokines. In SOD1(G93A) mouse model enhanced motor performance, decreased motor neuron cell death, and increased life span [69].
**Olesoxime**	[70]	MItochondrial pore modulator. Sunyach et al. [71] using SOD1(G93A) mice showed that it delays disease onset and improves survival by slowing muscle denervation, microglial activation, astrogliosis and MN death.
**Rasagiline**	(NCT01879241), (NCT01786603)	Inhibitor of monoamine oxidase B, antioxidant and anti-apoptotic functions. Presented significant dose-dependent therapeutic effect on motor function and survival of the animals [72].
**Pioglitazone**	[73]	Peroxisome proliferator-activated receptor gama agonist acting as potent anti-inflammatory and neuroprotective agent.<br><br>Treated SOD1(G93A) tg mice revealed improved muscle strength, delayed disease onset, and tretated animals survived significantly longer.
**Pimozide**	[74]	The drug is safe and well tolerated while a phase II randomized, placebo controlled, double-blinded, multi-centre clinical trial has not been finished yet.

Table S5: Recent compounds (or approaches) with current interest and on going trials [48]. They do not show direct connection with SOD1 aggregation but were firstly used in SOD1-associated ALS animal models.

Compound	Reference and year	(speculated) mechanism /comments
**Masitinib**	[75]	Tyrosine kinase inhibitor exerts neuroprotection and immunomodulation by affecting microglia, macrophage, and mast cell activity in central and peripheral nervous systems [48]. In SOD1(G93A) mouse model extended survival post paralysis [76]. Long-term overall survival data [77] suggest that it could prolong survival by over 2 years if treatment starts prior to severe impairment of functionality.
**Induced pluripotent stem cells (iPS)**	Baloh et al. [78] reviews some examples of small scale clinical trials	Indirect applications in drug discovery for ALS [79,80].<br><br>Direct application aims to autologous stem cell transplantation therapies [9] and includes mesenchymal stem cells, immune cells, fetal neuron progenitor cells [78].
**Bosutinib**	[79]	Src/c-Abl inhibitor. In SOD1(G93A) mice delayed disease onset and increased modestly their life span [81].
**Ibudilast**	[82]	Anti-inflammatory role by affecting macrophages, and glial cells.
**Ambroxol**	[83]	β-glucocerebrosidace 2 (GBA2) inhibitor for the treatment of respiratory tract infectious disorders. In SOD1(G85R) tg mouse model deleayed disease onset, improved motor function, and rescued MN death by regulating the glycosphingolipid metabolism.

Table S6: Glide and Induced Fit Docking (IFD) theoretical scores[a] (kcal/mol) of the 12 LOPAC® molecules interacting with SOD1(A4V) in different binding pockets.

	**Pocket 1**		**Pocket 2**		**Pocket 3**		**Pocket 4**	
**LIGANDS**	**Docking Score**	**IFD Score**	**Docking Score**	**IFD Score**	**Docking Score**	**IFD Score**	**Docking Score**	**IFD Score**
Aurothioglucose	**-5.624**	**-653.63**	-4.255	-649.16	-5.486	-652.71	-4.344	-650.1
Bexarotene	**-4.874**	**-660.49**	-3.34	-652.18	-3.907	-654.52	-3.38	-653.1
Bosutinib	**-4.987**	**-659.3**	-3.425	-653.43	-3.484	-657.56	-2.776	-657.2
Calcimycin	**-5.579**	**-660.86**	-3.355	-653.88	-3.584	-652.50	-3.383	-651.2
Cephalosporin C	**-5.443**	**-663.16**	-3.612	-658.33	-5.017	-660.21	-4.059	-659.2
Cyclosporine	-5.251	-	-3.169	-	-	-	-1.809	-
Diacylglycerol kinase inhibitor II	**-4.822**	**-658.62**	-2.357	-653.48	-3.177	-655.17	-2.071	-654.6
Ganciclovir	-5.937	-656.09	-4.655	-654.35	**-6.010**	**-656.13**	-3.836	-654
Icaritin	**-5.472**	**-658.77**	-2.969	-650.07	-4.660	-654.27	-	-654.4
N,N-Dihexyl-2-(4-fluorophenyl)indole-3-acetamide	**-4.345**	**-659.64**	-2.552	-651.31	-2.932	-654.67	-2.051	-654.4
Rabeprazole	**-5.99**	**-663.72**	-4.944	-654.88	-5.201	-656.83	-4.123	-656.2
Theophylline	**-6.318**	**-654.48**	-5.423	-651.89	-5.378	-654.79	-4.844	-650.5

[a]The lowest energy structure scores of each ligand are shown in the table.

References

1. Broom, H. R. *et al.* Folding and Aggregation of Cu, Zn-Superoxide Dismutase. *Amyotrophic Lateral Sclerosis* (2012).
2. Shaw, B. & Valentine, J. How do ALS-associated mutations in superoxide dismutase 1 promote aggregation of the protein? *Trends in Biochemical Sciences* **32**, 78–85 (2007).
3. Berry, J. D. *et al.* Design and Initial Results of a Multi-Phase Randomized Trial of Ceftriaxone in Amyotrophic Lateral Sclerosis. *PLoS ONE* **8**, e61177 (2013).
4. Cudkowicz, M. E. *et al.* Safety and efficacy of ceftriaxone for amyotrophic lateral sclerosis: a multi-stage, randomised, double-blind, placebo-controlled trial. *The Lancet Neurology* **13**, 1083–1091 (2014).
5. Guo, H. Increased expression of the glial glutamate transporter EAAT2 modulates excitotoxicity and delays the onset but not the outcome of ALS in mice. *Human Molecular Genetics* **12**, 2519–2532 (2003).
6. Rothstein, J. D. *et al.* β-Lactam antibiotics offer neuroprotection by increasing glutamate transporter expression. *Nature* **433**, 73–77 (2005).
7. Pascuzzi, R. M. *et al.* A phase II trial of talampanel in subjects with amyotrophic lateral sclerosis. *Amyotrophic Lateral Sclerosis* **11**, 266–271 (2010).
8. Chen, P.-C., Hsieh, Y.-C., Huang, C.-C. & Hu, C.-J. Tamoxifen for amyotrophic lateral sclerosis: A randomized double-blind clinical trial. *Medicine* **99**, e20423 (2020).
9. Wijesekera, L. C. & Leigh, P. N. Amyotrophic lateral sclerosis. *Orphanet J Rare Dis* **4**, 3 (2009).
10. Gordon, P. H. *et al.* Efficacy of minocycline in patients with amyotrophic lateral sclerosis: a phase III randomised trial. *Lancet Neurol* **6**, 1045–1053 (2007).

11. Gordon, P. H. *et al.* Placebo-controlled phase I/II studies of minocycline in amyotrophic lateral sclerosis. *Neurology* **62**, 1845–1847 (2004).

12. Van Den Bosch, L., Tilkin, P., Lemmens, G. & Robberecht, W. Minocycline delays disease onset and mortality in a transgenic model of ALS: *Neuroreport* **13**, 1067–1070 (2002).

13. Zhu, S. *et al.* Minocycline inhibits cytochrome c release and delays progression of amyotrophic lateral sclerosis in mice. *Nature* **417**, 74–78 (2002).

14. Kriz, J., Nguyen, M. D. & Julien, J.-P. Minocycline Slows Disease Progression in a Mouse Model of Amyotrophic Lateral Sclerosis. *Neurobiology of Disease* **10**, 268–278 (2002).

15. Miller, R. *et al.* Phase II/III randomized trial of TCH346 in patients with ALS. *Neurology* **69**, 776–784 (2007).

16. Kaufmann, P. *et al.* Phase II trial of CoQ10 for ALS finds insufficient evidence to justify phase III. *Ann Neurol.* **66**, 235–244 (2009).

17. Matthews, R. T., Yang, L., Browne, S., Baik, M. & Beal, M. F. Coenzyme Q10 administration increases brain mitochondrial concentrations and exerts neuroprotective effects. *Proc. Natl. Acad. Sci. U.S.A.* **95**, 8892–8897 (1998).

18. Desnuelle, C., Dib, M., Garrel, C. & Favier, A. A double-blind, placebo-controlled randomized clinical trial of α-tocopherol (vitamin E) in the treatment of amyotrophic lateral sclerosis. *Amyotrophic Lateral Sclerosis and Other Motor Neuron Disorders* **2**, 9–18 (2001).

19. Graf, M. *et al.* High dose vitamin E therapy in amyotrophic lateral sclerosis as add-on therapy to riluzole: results of a placebo-controlled double-blind study. *J Neural Transm* **112**, 649–660 (2005).

20. Ghadge, G. D. *et al.* Mutant superoxide dismutase-1-linked familial amyotrophic lateral sclerosis: molecular mechanisms of neuronal death and protection. *J Neurosci* **17**, 8756–8766 (1997).

21. Gurney, M. E. *et al.* Benefit of vitamin E, riluzole, and gababapentin in a transgenic model of familial amyotrophic lateral sclerosis. *Ann Neurol.* **39**, 147–157 (1996).

22. Cudkowicz, M. E. *et al.* Trial of celecoxib in amyotrophic lateral sclerosis. *Ann Neurol.* **60**, 22–31 (2006).

23. Drachman, D. B. *et al.* Cyclooxygenase 2 inhibition protects motor neurons and prolongs survival in a transgenic mouse model of ALS. *Ann Neurol.* **52**, 771–778 (2002).

24. Jan Groeneveld, G. *et al.* A randomized sequential trial of creatine in amyotrophic lateral sclerosis. *Ann Neurol.* **53**, 437–445 (2003).

25. Shefner, J. M. *et al.* A clinical trial of creatine in ALS. *Neurology* **63**, 1656–1661 (2004).

26. Klivenyi, P. *et al.* Neuroprotective effects of creatine in a transgenic animal model of amyotrophic lateral sclerosis. *Nat Med* **5**, 347–350 (1999).

27. Meininger, V. *et al.* Glatiramer acetate has no impact on disease progression in ALS at 40 mg/day: A double- blind, randomized, multicentre, placebo-controlled trial. *Amyotrophic Lateral Sclerosis* **10**, 378–383 (2009).

28. Angelov, D. N. *et al.* Therapeutic vaccine for acute and chronic motor neuron diseases: Implications for amyotrophic lateral sclerosis. *Proc. Natl. Acad. Sci. U.S.A.* **100**, 4790–4795 (2003).

29. Beauverd, M., Mitchell, J. D., Wokke, J. H. & Borasio, G. D. Recombinant human insulin-like growth factor I (rhIGF-I) for the treatment of amyotrophic

lateral sclerosis/motor neuron disease. *Cochrane Database of Systematic Reviews* (2012) doi:10.1002/14651858.CD002064.pub3.

30. Wen, D. *et al.* The role of insulin-like growth factor 1 in ALS cell and mouse models: A mitochondrial protector. *Brain Research Bulletin* **144**, 1–13 (2019).
31. Ochs, G. *et al.* A phase I/II trial of recombinant methionyl human brain derived neurotrophic factor administered by intrathecal infusion to patients with amyotrophic lateral sclerosis. *Amyotrophic Lateral Sclerosis and Other Motor Neuron Disorders* **1**, 201–206 (2000).
32. Meininger, V. *et al.* Efficacy and safety of xaliproden in amyotrophic lateral sclerosis: results of two phase III trials. *Amyotrophic Lateral Sclerosis and Other Motor Neuron Disorders* **5**, 107–117 (2004).
33. ALS CNTF Treatment Study Group. A double-blind placebo-controlled clinical trial of subcutaneous recombinant human ciliary neurotrophic factor (rHCNTF) in amyotrophic lateral sclerosis. *Neurology* **46**, 1244–1244 (1996).
34. de Paulis, T. ONO-2506. Ono. *Curr Opin Investig Drugs* **4**, 863–867 (2003).
35. Piepers, S. *et al.* Randomized sequential trial of valproic acid in amyotrophic lateral sclerosis. *Ann Neurol.* **66**, 227–234 (2009).
36. Sugai, F. *et al.* Benefit of valproic acid in suppressing disease progression of ALS model mice: Benefit of valproic acid in ALS. *European Journal of Neuroscience* **20**, 3179–3183 (2004).
37. Boll, M.-C. *et al.* Clinical and biological changes under treatment with lithium carbonate and valproic acid in sporadic amyotrophic lateral sclerosis. *Journal of the Neurological Sciences* **340**, 103–108 (2014).
38. Boll, M.-C., Alcaraz-Zubeldia, M., Rios, C., González-Esquivel, D. & Montes, S. A phase 2, double-blind, placebo-controlled trial of a valproate/lithium

combination in ALS patients. *Neurología (English Edition)* S217358082200089X (2022) doi:10.1016/j.nrleng.2022.07.003.

39. Feng, H.-L. *et al.* Combined lithium and valproate treatment delays disease onset, reduces neurological deficits and prolongs survival in an amyotrophic lateral sclerosis mouse model. *Neuroscience* **155**, 567–572 (2008).
40. Chio, A. *et al.* Lithium carbonate in amyotrophic lateral sclerosis: Lack of efficacy in a dose-finding trial. *Neurology* **75**, 619–625 (2010).
41. Shefner, J. *et al.* Safety, tolerability and pharmacodynamics of a skeletal muscle activator in amyotrophic lateral sclerosis. *Amyotrophic Lateral Sclerosis* **13**, 430–438 (2012).
42. Shefner, J. M. *et al.* A phase III trial of *tirasemtiv* as a potential treatment for amyotrophic lateral sclerosis. *Amyotrophic Lateral Sclerosis and Frontotemporal Degeneration* **20**, 584–594 (2019).
43. Gribkoff, V. K. & Bozik, M. E. KNS-760704 [(6R)-4,5,6,7-tetrahydro-N6-propyl-2, 6-benzothiazole-diamine dihydrochloride monohydrate] for the Treatment of Amyotrophic Lateral Sclerosis. *CNS Neuroscience & Therapeutics* **14**, 215–226 (2008).
44. Cudkowicz, M. *et al.* The effects of dexpramipexole (KNS-760704) in individuals with amyotrophic lateral sclerosis. *Nat Med* **17**, 1652–1656 (2011).
45. Cudkowicz, M. E. *et al.* Dexpramipexole versus placebo for patients with amyotrophic lateral sclerosis (EMPOWER): a randomised, double-blind, phase 3 trial. *The Lancet Neurology* **12**, 1059–1067 (2013).
46. Kaji, R. *et al.* Effect of ultrahigh-dose methylcobalamin on compound muscle action potentials in amyotrophic lateral sclerosis: A double-blind controlled study. *Muscle Nerve* **21**, 1775–1778 (1998).

47. Oki, R. *et al.* Efficacy and Safety of Ultrahigh-Dose Methylcobalamin in Early-Stage Amyotrophic Lateral Sclerosis: A Randomized Clinical Trial. *JAMA Neurol* **79**, 575 (2022).
48. Ketabforoush, A. H. M. E. *et al.* Masitinib: The promising actor in the next season of the Amyotrophic Lateral Sclerosis treatment series. *Biomedicine & Pharmacotherapy* **160**, 114378 (2023).
49. Cudkowicz, M. E. *et al.* A randomized, placebo-controlled trial of topiramate in amyotrophic lateral sclerosis. *Neurology* **61**, 456–464 (2003).
50. Cudkowicz, M. E. *et al.* Phase 2 study of sodium phenylbutyrate in ALS. *Amyotrophic Lateral Sclerosis* **10**, 99–106 (2009).
51. Ryu, H. *et al.* Sodium phenylbutyrate prolongs survival and regulates expression of anti-apoptotic genes in transgenic amyotrophic lateral sclerosis mice: Histone deacetylase therapy in amyotrophic lateral sclerosis. *Journal of Neurochemistry* **93**, 1087–1098 (2005).
52. Zinman, L., Sadeghi, R., Gawel, M., Patton, D. & Kiss, A. Are statin medications safe in patients with ALS? *Amyotrophic Lateral Sclerosis* **9**, 223–228 (2008).
53. Scelsa, S. N. *et al.* A pilot, double-blind, placebo-controlled trial of indinavir in patients with ALS. *Neurology* **64**, 1298–1300 (2005).
54. Miller, R. G. *et al.* A clinical trial of verapamil in amyotrophic lateral sclerosis. *Muscle Nerve* **19**, 511–515 (1996).
55. Zhang, X. *et al.* Verapamil Ameliorates Motor Neuron Degeneration and Improves Lifespan in the SOD1G93A Mouse Model of ALS by Enhancing Autophagic Flux. *Aging and disease* **10**, 1159 (2019).
56. Miller, R. G. *et al.* Controlled trial of nimodipine in amyotrophic lateral sclerosis. *Neuromuscular Disorders* **6**, 101–104 (1996).

57. The Italian ALS Study Group. Branched-chain amino acids and amyotrophic lateral sclerosis: A treatment failure? *Neurology* **43**, 2466–2466 (1993).

58. Caroscio, J. T. *et al.* A double-blind, placebo-controlled trial of TRH in amyotrophic lateral sclerosis. *Neurology* **36**, 141–141 (1986).

59. Morris, F. H., Denys, E. H. & Fallat, R. J. Trial of octacosanol in amyotrophic lateral sclerosis. *Neurology* **36**, 1263–1263 (1986).

60. Olson, W. H., Simons, J. A. & Halaas, G. W. Therapeutic trial of tilorone in ALS: Lack of benefit in a double-blind, placebo-controlled study. *Neurology* **28**, 1293–1293 (1978).

61. Fareed, G. C. & Tyler, H. R. The use of isoprinosine in patients with amyotrophic lateral sclerosis. *Neurology* **21**, 937–937 (1971).

62. Appel, S. H. *et al.* A Double-blind Study of the Effectiveness of Cyclosporine in Amyotrophic Lateral Sclerosis. *Archives of Neurology* **45**, 381–386 (1988).

63. Keep, M., Elmér, E., Fong, K. S. K. & Csiszar, K. Intrathecal cyclosporin prolongs survival of late-stage ALS mice. *Brain Research* **894**, 327–331 (2001).

64. Miller, R. G. *et al.* Phase III randomized trial of gabapentin in patients with amyotrophic lateral sclerosis. *Neurology* **56**, 843.1-848 (2001).

65. Kalra, S., Cashman, N. R., Caramanos, Z., Genge, A. & Arnold, D. L. Gabapentin therapy for amyotrophic lateral sclerosis: lack of improvement in neuronal integrity shown by MR spectroscopy. *AJNR Am J Neuroradiol* **24**, 476–480 (2003).

66. Meininger, V. *et al.* Pentoxifylline in ALS: A double-blind, randomized, multicenter, placebo-controlled trial. *Neurology* **66**, 88–92 (2006).

67. Morimoto, S. *et al. Ropinirole Hydrochloride for Amyotrophic Lateral Sclerosis: A Single-Center, Randomized Feasibility, Double-Blind, Placebo-Controlled*

Trial. http://medrxiv.org/lookup/doi/10.1101/2021.12.05.21267266 (2021) doi:10.1101/2021.12.05.21267266.

68. Stommel, E. W. *et al.* Efficacy of thalidomide for the treatment of amyotrophic lateral sclerosis: A phase II open label clinical trial. *Amyotrophic Lateral Sclerosis* **10**, 393–404 (2009).
69. Kiaei, M. *et al.* Thalidomide and Lenalidomide Extend Survival in a Transgenic Mouse Model of Amyotrophic Lateral Sclerosis. *J. Neurosci.* **26**, 2467–2473 (2006).
70. Lenglet, T. *et al.* A phase II−III trial of olesoxime in subjects with amyotrophic lateral sclerosis. *Eur J Neurol* **21**, 529–536 (2014).
71. Sunyach, C. *et al.* Olesoxime delays muscle denervation, astrogliosis, microglial activation and motoneuron death in an ALS mouse model. *Neuropharmacology* **62**, 2346–2353 (2012).
72. Waibel, S., Reuter, A., Malessa, S., Blaugrund, E. & Ludolph, AlbertC. Rasagiline alone and in combination with riluzole prolongs survival in an ALS mouse model. *J Neurol* **251**, (2004).
73. Dupuis, L. *et al.* A Randomized, Double Blind, Placebo-Controlled Trial of Pioglitazone in Combination with Riluzole in Amyotrophic Lateral Sclerosis. *PLoS ONE* **7**, e37885 (2012).
74. Patten, S. A. *et al.* Neuroleptics as therapeutic compounds stabilizing neuromuscular transmission in amyotrophic lateral sclerosis. *JCI Insight* **2**, e97152 (2017).
75. Mora, J. S. *et al.* Masitinib as an add-on therapy to riluzole in patients with amyotrophic lateral sclerosis: a randomized clinical trial. *Amyotrophic Lateral Sclerosis and Frontotemporal Degeneration* **21**, 5–14 (2020).

76. Trias, E. *et al.* Post-paralysis tyrosine kinase inhibition with masitinib abrogates neuroinflammation and slows disease progression in inherited amyotrophic lateral sclerosis. *J Neuroinflammation* **13**, 177 (2016).

77. Mora, J. S. *et al.* Long-term survival analysis of masitinib in amyotrophic lateral sclerosis. *Ther Adv Neurol Disord* **14**, 175628642110303 (2021).

78. Baloh, R. H., Glass, J. D. & Svendsen, C. N. Stem cell transplantation for amyotrophic lateral sclerosis. *Current Opinion in Neurology* **31**, 655–661 (2018).

79. Imamura, K. *et al.* Safety and tolerability of bosutinib in patients with amyotrophic lateral sclerosis (iDReAM study): A multicentre, open-label, dose-escalation phase 1 trial. *eClinicalMedicine* **53**, 101707 (2022).

80. Okano, H., Yasuda, D., Fujimori, K., Morimoto, S. & Takahashi, S. Ropinirole, a New ALS Drug Candidate Developed Using iPSCs. *Trends in Pharmacological Sciences* **41**, 99–109 (2020).

81. Imamura, K. *et al.* The Src/c-Abl pathway is a potential therapeutic target in amyotrophic lateral sclerosis. *Sci. Transl. Med.* **9**, eaaf3962 (2017).

82. Oskarsson, B. *et al.* MN-166 (ibudilast) in amyotrophic lateral sclerosis in a Phase IIb/III study: COMBAT-ALS study design. *Neurodegenerative Disease Management* **11**, 431–443 (2021).

83. Shah, S., Dooms, M. M., Amaral-Garcia, S. & Igoillo-Esteve, M. Current Drug Repurposing Strategies for Rare Neurodegenerative Disorders. *Front. Pharmacol.* **12**, 768023 (2021).
